# Comparison of Ten Surrogate Insulin Resistance and Obesity Markers to Identify Metabolic Syndrome in Mexican Adults

**DOI:** 10.3390/metabo14070358

**Published:** 2024-06-26

**Authors:** Iván Filiberto Contreras-Hernández, Cruz Vargas-De-León, Luis Rey García-Cortes, Adriana Flores-Miranda, Rodrigo Romero-Nava, María Esther Ocharán-Hernández

**Affiliations:** 1 Laboratorio de Modelación Bioestadística para la Salud, Sección de Estudios de Posgrado e Investigación, Escuela Superior de Medicina, Instituto Politécnico Nacional, Ciudad de México 11340, Mexico; drivancondez@gmail.com (I.F.C.-H.); rromeron@ipn.mx (R.R.-N.); estherocharan@hotmail.com (M.E.O.-H.); 2 IMSS Unidad de Medicina Familiar 75, Delegación 15 Oriente, Instituto Mexicano del Seguro Social, Estado de México 57500, Mexico; 3 División de Investigación, Hospital Juárez de México, Ciudad de México 07760, Mexico; afloresm.124@gmail.com; 4 Jefatura de Servicios de Prestaciones Médicas, Órgano de Operación Administrativa Desconcentrada Regional Estado de México Oriente, Instituto Mexicano del Seguro Social, Estado de México 54060, Mexico; luis.garciaco@imss.gob.mx

**Keywords:** metabolic syndrome, insulin resistance, obesity, hyperglycemia, dyslipidemia, hypertension, ATP III, IDF

## Abstract

Metabolic syndrome (*MetS*) is a group of clinical traits directly linked to type 2 diabetes mellitus and cardiovascular diseases, whose prevalence has been rising nationally and internationally. We aimed to evaluate ten known and novel surrogate markers of insulin resistance and obesity to identify *MetS* in Mexican adults. The present cross-sectional study analyzed 10575 participants from ENSANUT-2018. The diagnosis of *MetS* was based on the Adult Treatment Panel III (ATP III) criteria and International Diabetes Federation (IDF) criteria, stratified by sex and age group. According to ATP III, the best biomarker was the metabolic score for insulin resistance (METS-IR) in men aged 20–39 and 40–59 years and lipid accumulation product (LAP) in those aged ≥60 years. The best biomarker was LAP in women aged 20–39 and triglyceride–glucose index (TyG) in those aged 40–59 and ≥60 years. Using the IDF criteria, the best biomarker was LAP in men of all ages. TyG gave the best results in women of all ages. The best biomarker for diagnosis of *MetS* in Mexican adults depends on the criteria, including sex and age group. LAP and TyG are easy to obtain, inexpensive, and especially useful at the primary care level.

## 1. Introduction

Metabolic syndrome (*MetS*) is a set of related clinical characteristics that include central obesity, hypertension, hyperglycemia, and atherogenic dyslipidemia (low HDL cholesterol and hypertriglyceridemia). It is a clinical entity directly related to chronic diseases that cause an increase in morbidity, mainly type 2 diabetes mellitus (DMT2) and cardiovascular diseases (CVDs) [1,2,3]. Cardiovascular diseases are one of the leading causes of death worldwide [4], and it is estimated that just over 10.5% of the world’s population suffers from DMT2 [5].

In Mexico, for years, heart diseases, especially ischemic diseases, have been among the leading causes of death [6]. In fact, deaths related to these diseases increased from 9.2 in 2011 to 17.9 per 10,000 inhabitants in 2021 [7]. According to data from 2018, the prevalence of *MetS* has increased by 16.75% (IC95%, according to Adult Treatment Panel III criteria (ATP III)) and 41.88% (IC95%, under the International Diabetes Federation (IDF) criteria) [8].

The two conditions that have been confirmed as closely related to *MetS* are obesity and insulin resistance (IR). The relationship between increased subcutaneous adipose tissue and the development of a proinflammatory state has been studied. In addition, the contribution of increased fat tissue to the development of IR is known [9,10,11,12].

There are various sets of criteria for the definition of *MetS*. The best accepted and used are the IDF [13] and ATP III criteria [14]. These criteria are applicable at the secondary and tertiary care levels. For this reason, other indices or markers have been explored to diagnose *MetS* at the primary care level, where some fasting biochemical parameters are not available to make the diagnosis. The traditional indicators of obesity, such as body mass index (BMI) and waist circumference (WC), have been used to diagnose *MetS* but have shown poor diagnostic performance with sensitivity or specificity below 75% [15], and they are also of limited utility for identifying subclinical conditions [16]. Recently, different surrogate markers for a simple, affordable, and inexpensive *MetS* diagnosis have been studied.

Some of these markers have been analyzed in Asian countries. Studies in China [17] and Bangladesh [18] found TyG to be one of the best surrogate markers for determining *MetS*. However, they also found LAP and the visceral adiposity index (VAI) to be efficacious in middle-aged and elderly Chinese [18]. The results were similar to those obtained in the Korean population [19]. TyG was a better marker of *MetS* in India [20]; however, in Taiwan, it was determined that the VAI as a marker was more accurate but difficult to access [15].

One US study proposed a new measurement index, the body roundness index (BRI), to assess individual body fat distribution and overall adiposity. It was introduced as an alternative to traditional measures of obesity, such as BMI, which may not capture variations in body fat distribution. BRI takes into account both WC and hip circumference (HC) to provide a more comprehensive assessment of body shape, and it is a good predictor of body fat percentage [21]. On the other hand, a Colombian study that evaluated diabetes predictors such as BMI, WC, waist-to-height ratio (WtHR), triglyceride-to-glucose fasting related to BMI (TyG-BMI), triglyceride-to-glucose fasting related to WC (TyG-WC), and triglyceride-to-glucose fasting related to WtHR (TyG-WtHR) concluded that TyG was the best indicator in the adult population [22].

A cross-sectional study conducted on adults from the center of Mexico considered only the triglyceride/high-density cholesterol (TG/HDL) ratio [23]. No other studies have been conducted in Mexico to identify the best markers of insulin resistance and obesity to improve the diagnosis of metabolic syndrome in adults, considering fasting blood chemistry parameters at the primary care level. Therefore, the present project aims to evaluate (known and novel) surrogate markers of insulin resistance and obesity to diagnose *MetS* in Mexican adults.

## 2. Materials and Methods

### 2.1. Study Population

This analysis was carried out based on the information obtained from the National Health and Nutrition Survey (ENSANUT, for its acronym in Spanish) 2018 and was designed to quantify the frequency and distribution of health and nutrition conditions of the Mexican population. The ENSANUT 2018 had a transversal, probabilistic, multi-stage, and cluster sampling design, with regional representation (north, center, Mexico City, and south) and considered urban (population ≥ 2500 inhabitants) and rural (population < 2500 inhabitants) locality. Information was obtained from 50654 households distributed in the 32 states of the country. When considering selecting individuals (one for each age group per household), 43,078 adults aged 20 or older were interviewed. A detailed description of the sampling procedures and survey methodology has already been published [24].

For this study, the inclusion criteria were Mexican men and women over 20 years of age. The exclusion criteria were serum glucose concentrations of less than 70 mg/dL or greater than 500 mg/dL, serum triglycerides greater than 1200 mg/dL, and a height of less than 1.30 m, while the elimination criteria were missing information on body composition and biochemical indices.

### 2.2. Definition of Metabolic Syndrome

The diagnosis of *MetS* was based on the IDF [13] and ATP III [14] definitions. The IDF diagnoses *MetS* when patients present a waist circumference of ≥90 cm in men or ≥80 cm in women, plus two of the following conditions: triglycerides ≥ 150 mg/dL or medication treatment to control triglycerides; high-density cholesterol < 40 mg/dL in men and < 50 mg/dL in women; blood pressure ≥ 130/85 mmHg or previous diagnosis of hypertension; fasting glucose ≥100 mg/dL or previous diagnosis of diabetes. ATP III establishes the presence of MetS when three or more of the following findings occur: waist circumference ≥ 102 cm in men or ≥88 cm in women; fasting glucose ≥ 110 mg/dL or previous diagnosis of diabetes; triglycerides ≥ 150 mg/dL; blood pressure ≥ 130/85 mmHg or previous diagnosis of hypertension; high-density lipoprotein cholesterol < 40 mg/dL in men and <50 mg/dL in women. The diagnostic criteria of ATP III and the IDF are shown in Table A1.

### 2.3. Surrogate Markers of Insulin Resistance and Obesity

Surrogate markers of insulin resistance and obesity include body shape index (ABSI), BRI, LAP, the metabolic score for insulin resistance (METS-IR), the metabolic score for visceral fat (METS-VF), single-point insulin sensitivity estimator (SPISE), TG/HDL, TyG, VAI, and visceral adipose tissue (VAT). They were calculated using the following formulas: SPISE [25], LAP [26], VAI [27], TyG [28], TG/HDL [29], METS-IR [30], METS-VF [31], and VAT [31]:

SPISE = (600 × HDL (mg/dL)^0.185^)/(Tri (mg/dL))^0.2^ × (BMI)^1.338^ (1)

METS-IR = (ln [2 × Glu (mg/dL) + Tri (mg/dL)] × BMI (kg/m^2^))/ln[HDL (mg/dL)] (2)

TG/HDL = (Tri (mg/dL))/(HDL (mg/dL))(3)

TyG = [ln(Tri (mg/dL) × Glu (mg/dL))]/2(4)

LAP Women = (WC (cm) − 58) × Tri (mmol/L)(5)

LAP Men = (WC (cm) − 65) × Tri (mmol/L)(6)

VAI Women = [(WC (cm))/((1.89 × BMI (kg/m^2^)) + 36.58)] + [(Tri (mmol/L))/0.81] + [1.52/(HDL (mmol/L))] (7)

VAI Men = [(WC (cm))/((1.88 × BMI) + 39.68)] × [(Tri (mmol/L))/1.03] × [1.31/(HDL (mmol/L))](8)

METS-VF = 4.466 + 0.011 (ln(METS-IR))^3^ + 3.239 (ln(WHtR))^3^ + 0.319 (Sex) + 0.594 ln(Age) (9)

VAT = e^4.466 + 0.011 [(ln(METS-IR))^3^] + 3.239 [(ln(WHtR))^3^] + 0.319 (Sex) + 0.594 (ln(Age))^ (10)

where Sex = 1 (0) if man (woman), and WHtR denotes waist-to-height ratio.

Participants were stratified by sex and age group (20–39 years, 40–59 years, and ≥60 years), which were compared with respect to the following markers of insulin resistance and obesity: SPISE, LAP, VAI, TyG, TG/HDL ratio, METS-IR, METS-VF, and VAT.

### 2.4. Statistical Analysis

All normally distributed data are presented as mean (standard deviation), and non-normally distributed are presented as median (25th (Q1) and 75th (Q3) quartiles) for numerical variables and counts (percentage, %) for categorical variables. Receiver operating characteristic (ROC) curves were generated to calculate areas under the curve (AUC), determining the cut-off value for each marker and selecting the one with the highest Youden index (YI) as the best one.

The following accuracy measures and their 95% confidence intervals are reported: sensitivity, specificity, positive and negative predictive values, positive and negative likelihood ratio, and the YI. The YI was calculated by adding the sensitivity, with the specificity – 1 being the maximum value reached within the ROC curve. Confidence intervals for sensitivity, specificity, and positive and negative predictive values were computed with the “exact” Clopper–Pearson confidence intervals. Using the Bootstrap method, we implemented a simple code in the R software 4.4.1 to calculate the confidence intervals of the Youden index.

To compare the areas under the curve (AUC) between the indices, we used the bootstrap test for two correlated ROC curves implemented in the “pROC” library of the R software 4.4.1 [32]. All statistical analyses were performed by using IBM SPSS Statistics 23.0 and R statistical software 4.4.1, whereas ROC curves were elaborated in IBM SPSS Statistics 23.0. Results were considered significant at *p* < 0.05 (2-sided).

## 3. Results

### 3.1. Characteristics of the Study Population

The ENSANUT-2018 database reported two databases, the anthropometric parameters database with 33,818 and the biochemical database with 13,220. To carry out the calculations of the different markers, these bases were joined; applying the elimination criteria, we have 13,101; of these, when applying the elimination criteria, there were 10,575.

**Figure 1:**
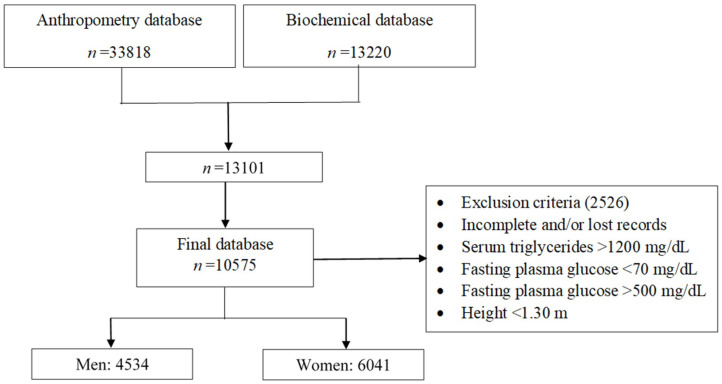
Flow diagram for obtaining the database for the diagnosis of *MetS*.

Two databases were created from the 10,575 database by sex: one with 6041 women and another with 4534 men. Each database was subdivided within it by age group (Figure 1). The database contained the following variables: age, weight, height, systolic and diastolic blood pressure, WC, total cholesterol, HDL cholesterol, triglycerides, glucose, and the dichotomous variables of treatment and diagnosis for diabetes, hypercholesterolemia, hypertriglyceridemia, and hypertension, respectively. The ATP III and IDF criteria were calculated using the variables from each database. The variables from the database were used to calculate the ten markers. Finally, each biomarker was evaluated for its discriminatory ability for each metabolic syndrome criterion.

The sociodemographic, anthropometric, and biochemical characteristics of the study population are presented in Table 1. The study population consisted of 10,575 participants, most of whom (57%) were women. The highest proportion of the study population was in the 40–59-year age group (38.7%). Concerning nutritional status measured by body mass index, the majority were obese (52.7%), and women had more significant central obesity measured by waist circumference in relation to the ATP III and IDF criteria, respectively (73.6% and 88.9%). Systolic blood pressure was above 135 mmHg in 34.6% of the population, while diastolic blood pressure was elevated in 20.7%. In terms of biochemical parameters, 33.6% of the participants had elevated cholesterol levels above 200 mg/dL; 40.9% and 76.5% of men and women had HDL cholesterol below 40 and 50 mg/dL in men and women, respectively; and 58.6% had triglycerides above 150 mg/dL. Based on the IDF or ATP III criteria, blood glucose concentrations above 100 mg/dL and 110 mg/dL were obtained in 30.4% and 19.1% of participants, respectively.

Of the total population of women, 52.6% and 60.0% presented *MetS* according to the ATP III and IDF criteria, respectively, while of the total population of men, it was 39.9% and 53.6%, respectively.

### 3.2. Diagnostic Indices for MetS According to the ATP III Criteria

**Figure 2:**
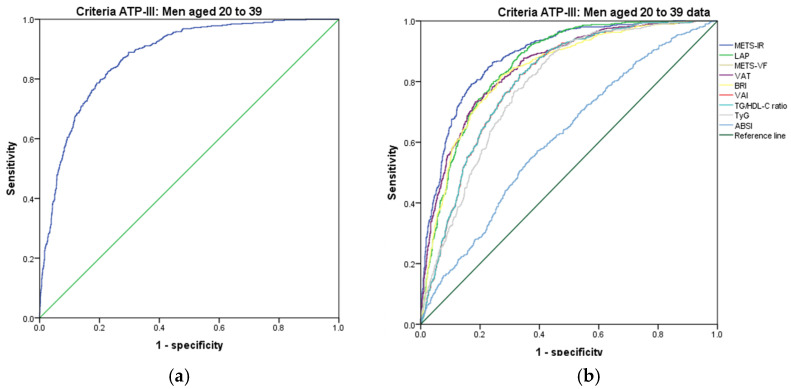
ROC curves for men aged 20–39 years according to the ATP III criteria, evaluating the following surrogate markers with the reference line: (**a**) SPISE; (**b**) METS-IR, LAP, METS-VF, VAT, BRI, VAI, TG/HDL-C ratio, TyG, and ABSI.

**Figure 3:**
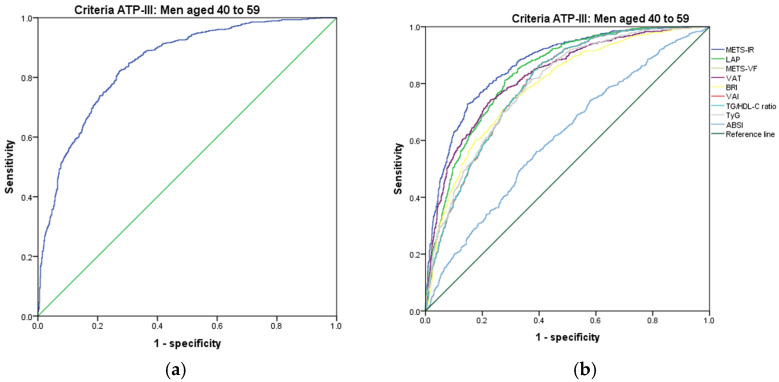
ROC curves for men aged 40–59 years according to the ATP III criteria, evaluating the following surrogate markers with the reference line: (**a**) SPISE; (**b**) METS-IR, LAP, METS-VF, VAT, BRI, VAI, TG/HDL-C ratio, TyG, and ABSI.

**Figure 4:**
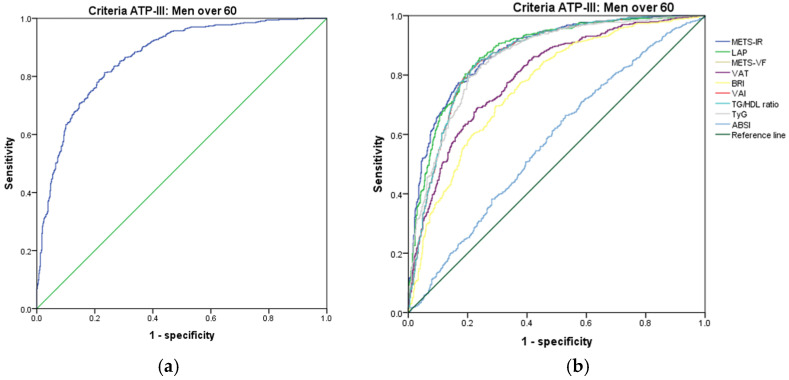
ROC curves for men over 60 according to the ATP III criteria, evaluating the following surrogate markers with the reference line: (**a**) SPISE; (**b**) METS-IR, LAP, METS-VF, VAT, BRI, VAI, TG/HDL-C ratio, TyG, and ABSI.

**Figure 5:**
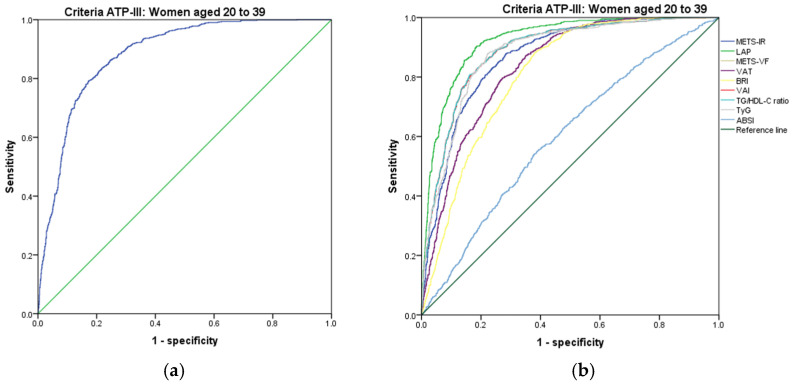
ROC curves for women aged 20–39 years according to the ATP III criteria, evaluating the following surrogate markers with the reference line: (**a**) SPISE; (**b**) METS-IR, LAP, METS-VF, VAT, BRI, VAI, TG/HDL-C ratio, TyG, and ABSI.

**Figure 6:**
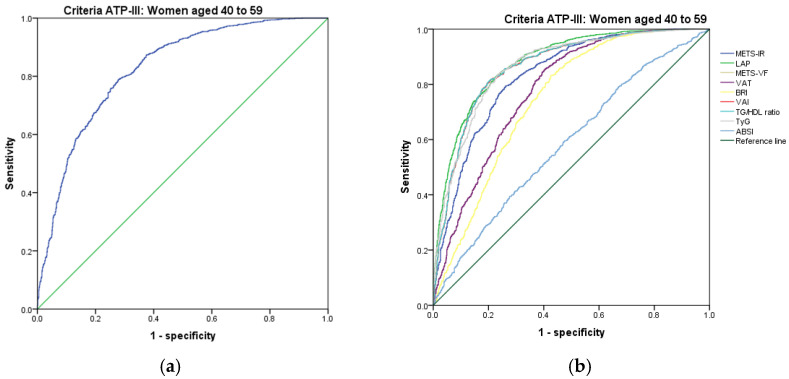
ROC curves for women aged 40–59 years according to the ATP III criteria, evaluating the following surrogate markers with the reference line: (**a**) SPISE; (**b**) METS-IR, LAP, METS-VF, VAT, BRI, VAI, TG/HDL-C ratio, TyG, and ABSI.

Figure 1, Figure 2, Figure 3, Figure 4, Figure 5 and Figure 6 compare the ROC curves, and Table 2, Table 3, Table 4, Table 5, Table 6 and Table 7 report the accuracy measures for the diagnosis of *MetS* according to the ATP III criteria using the following markers: ABSI, BRI, LAP, METS-IR, METS-VF, SPISE, TG/HDL ratio, TyG, VAI, and VAT.

In men aged 20–39, we found that METS-IR had a higher Youden index (AUC = 0.884 (95% CI 0.868–0.900); Se = 88.5 (95% CI 85.4–91.1); Sp = 71.3 (95% CI 68.6–73.4); YI = 59.8 (95% CI 56.0–63.6); and cut-off value ≥ 45.29). The following markers had lower diagnostic accuracy than METS-IR: SPISE (AUC = 0.875 (95% CI 0.858–0.892); Se = 67.8 (95% CI 63.5–71.8); Sp = 87.5 (95% CI 85.8–89.5); YI = 55.3 (95% CI 51.0–59.9); and cut-off value ≤4.13) and LAP (AUC = 0.855 (95% CI 0.837–0.873); Se = 85.5 (95% CI 82.2–88.5); Sp = 69.1 (95% CI 66.4–71.7); YI = 54.6 (95% CI 50.6–58.6); and cut-off value ≥ 65.22). See Figure 2 and Table 2. We found significant differences in the AUC for the diagnosis of *MetS* according to the ATP III criteria between the METS-IR and SPISE (*p* < 0.001) and between SPISE and LAP (*p* = 0.001). We chose the METS-IR to diagnose *MetS* according to the ATP III criteria in men aged 20–39 because it has a greater discriminating capacity.

In men aged 40–59, we found that METS-IR had a higher Youden index (AUC = 0.867 (95% CI 0.850–0.883); Se = 86.8 (95% CI 84.2–89.1); Sp = 68.0 (95% CI 64.9–70.9); YI = 54.8 (95% CI 50.9–58.6); and cut-off value ≥ 45.08). The following markers had lower diagnostic accuracy than METS-IR: LAP (AUC = 0.836 (95% CI 0.817–0.854); Se = 83.6 (95% CI 80.8–86.2); Sp = 68.8 (95% CI 65.8–71.8); YI = 52.4 (95% CI 48.3–56.3); and cut-off value ≥ 70.75) and SPISE (AUC = 0.846 (95% CI 0.828–0.865); Se = 64.1 (95% CI 60.5–67.5); Sp = 84.7 (95% CI 82.2–86.9); YI = 48.8 (95% CI 44.7–52.7); and cut-off value ≤ 4.22). See Figure 3 and Table 3. We found significant differences in the AUC of *MetS* diagnosis according to the ATP III criteria between the METS-IR and SPISE (*p* < 0.001), but there was no difference between LAP and SPISE (*p* = 0.119). We chose the METS-IR for diagnosis of *MetS* according to the ATP III criteria in men aged 40–59 because it has a greater discriminating capacity.

In men aged 60 and over, we found that LAP had a higher Youden index (AUC = 0.876 (95% CI 0.855–0.896); Se = 87.6 (95% CI 84.6–90.3); Sp = 73.2 (95% CI 69.4–76.8); YI = 60.8 (95% CI 56.8–65.8); and cut-off value ≥ 55.83). The following markers had lower diagnostic accuracy than LAP: TG/HDL ratio (AUC = 0.862 (95% CI 0.840–0.883); Se = 86.3 (95% CI 83.1–89.1); Sp = 72.9 (95% CI 69.0–76.5); YI = 59.2 (95% CI 54.7–64.3); and cut-off value ≥ 3.84) and VAI (AUC = 0.861 (95% CI 0.839–0.883); Se = 86.1 (95% CI 82.9–89.0); Sp = 72.9 (95% CI 69.0–76.5); YI = 59.0 (95% CI 54.1–63.6); and cut-off value ≥ 89.26). See Figure 4 and Table 4. We found no differences in the AUC for the diagnosis of *MetS* according to the ATP III criteria between LAP and the TG/HDL ratio (*p* = 0.111), between LAP and VAI (*p* = 0.116), and between the TG/HDL ratio and VAI (*p* = 0.114). We chose LAP to diagnose *MetS* according to the ATP III criteria in men 60 years of age or older. Although it has the same discrimination ability as the TG/HDL ratio, LAP only uses triglycerides and waist circumference.

In women aged 20–39, we found that LAP had a higher Youden index (AUC = 0.924 (95% CI 0.913–0.935); Se = 92.4 (95% CI 90.4–94.1); Sp = 78.1 (95% CI 75.9–80.2); YI = 70.5 (95% CI 67.6–73.2); and cut-off value ≥ 56.01). The following markers had lower diagnostic accuracy than LAP: TyG (AUC = 0.880 (95% CI 0.866–0.895); Se = 88.1 (95% CI 85.7–90.2); Sp = 77.1 (95% CI 74.9–79.3); YI = 65.2 (95% CI 62.0–68.2); and cut-off value ≥ 4.73) and TG/HDL ratio (AUC = 0.888 (95% CI 0.874–0.901); Se = 88.9 (95% CI 86.6–91.0); Sp = 74.9 (95% CI 72.6–77.1); YI = 63.8 (95% CI 60.6–66.8); and cut-off value ≥ 3.31). See Figure 5 and Table 5. We found significant differences in the AUC for the diagnosis of *MetS* according to the ATP III criteria between the LAP and TyG (*p* < 0.001). However, there was no difference between TyG and the TG/HDL ratio (*p* = 0.110). We chose the LAP to diagnose *MetS* according to the ATP III criteria in women aged 20–39 because it has a greater discriminating capacity.

In women aged 40–59, we found that TyG had a higher Youden index (AUC = 0.866 (95% CI 0.851–0.881); Se = 86.6 (95% CI 84.8–88.3); Sp = 71.7 (95% CI 68.7–74.6); YI = 58.3 (95% CI 54.9–61.6); and cut-off value ≥ 4.77). The following markers had lower diagnostic accuracy than TyG: TG/HDL ratio (AUC = 0.866 (95% CI 0.850–0.881); Se = 86.7 (95% CI 84.9–88.4); Sp = 71.4 (95% CI 68.3–74.3); YI = 58.1 (95% CI 54.6–61.5); and cut-off value ≥ 3.16) and LAP (AUC = 0.881 (95% CI 0.867–0.895); Se = 88.1 (95% CI 86.4–89.7); Sp = 69.9 (95% CI 66.8–72.8); YI = 58.0 (95% CI 54.6–61.3); and cut-off value ≥ 60.23). See Figure 6 and Table 6. We found no differences in the AUC for the diagnosis of *MetS* according to the ATP III criteria between TyG and the TG/HDL ratio (*p* = 0.941); however, there was a significant difference between the TG/HDL ratio and LAP (*p* = 0.028). We chose TyG to diagnose *MetS* according to the ATP III criteria in women aged 40–59. Although it has the same discrimination ability as the TG/HDL ratio, TyG only uses glucose and triglycerides.

**Figure 7:**
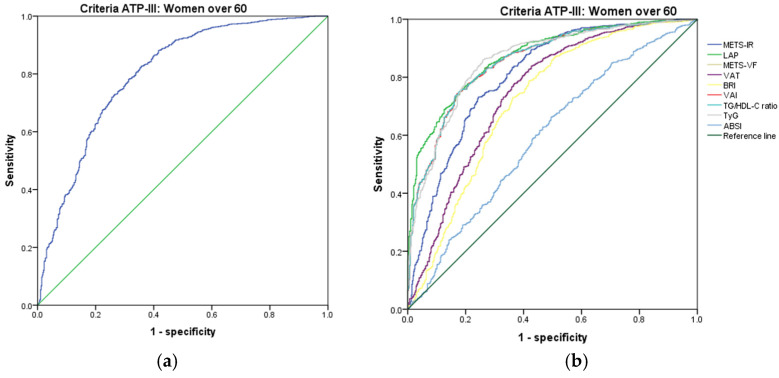
ROC curves for women over 60 years according to the ATP III criteria, evaluating the following surrogate markers with the reference line (**a**) SPISE; (**b**) METS-IR, LAP, METS-VF, VAT, BRI, VAI, TG/HDL-C ratio, TyG, and ABSI.

In women over 60 years, we found that TyG had a higher Youden index (AUC = 0.860 (95% CI 0.839–0.880); Se = 86.0 (95% CI 83.6–88.3); Sp = 74.0 (95% CI 69.8–77.8), YI = 60.0 (95% CI 55.3–64.5); and cut-off value ≥ 4.75). The following markers had lower diagnostic accuracy than TyG: the TG/HDL ratio (AUC = 0.857 (95% CI 0.836–0.877); Se = 85.8 (95% CI 83.3–88.0); Sp = 68.2 (95% CI 63.9–72.3); YI = 54.0 (95% CI 49.2–58.6); and cut-off value ≥ 2.84) and VAI (AUC = 0.856 (95% CI 0.836–0.877); Se = 85.7 (95% CI 83.2–87.9); Sp = 68.0 (95% CI 63.6–72.1); YI = 53.7 (95% CI 49.0–58.4); and cut-off value ≥ 89.27). See Figure 7 and Table 7. We found no differences in the AUC for the diagnosis of *MetS* according to the ATP III criteria between TyG and TG/HDL ratio (*p* = 0.696); however, there was a significant difference between TG/HDL ratio and VAI (*p* = 0.037). Similar to the previous age group, we chose TyG to diagnose *MetS* with the criteria of ATP III in women over 60 years.

### 3.3. Indices for the Diagnosis of MetS According to the IDF Criteria

**Figure 8:**
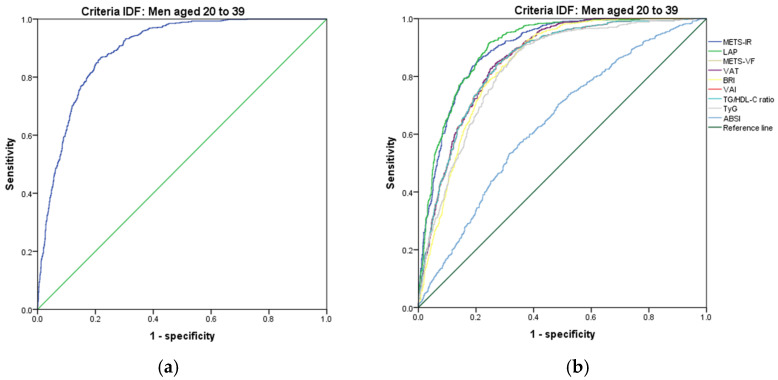
ROC curves for men aged 20–39 years according to the IDF criteria, evaluating the following surrogate markers with the reference line: (**a**) SPISE; (**b**) METS-IR, LAP, METS-VF, VAT, BRI, VAI, TG/HDL-C ratio, TyG, and ABSI.

**Figure 9:**
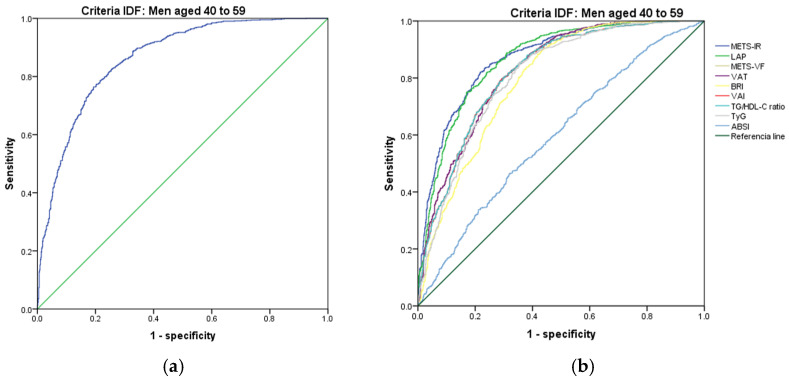
ROC curves for men aged 40–59 years according to the IDF criteria, evaluating the following surrogate markers with the reference line: (**a**) SPISE; (**b**) METS-IR, LAP, METS-VF, VAT, BRI, VAI, TG/HDL-C ratio, TyG, and ABSI.

**Figure 10:**
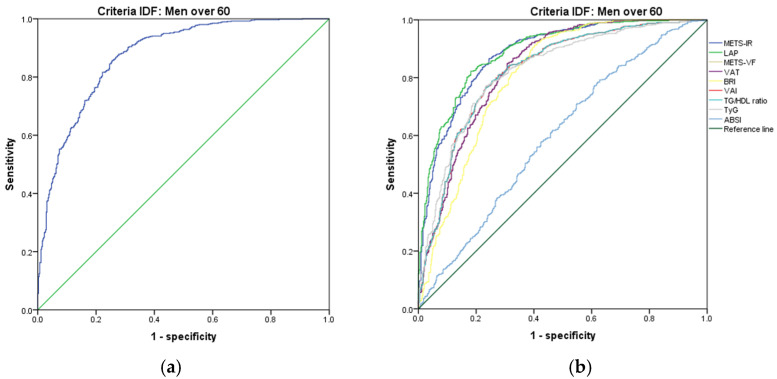
ROC curves for men over 60 according to the IDF criteria, evaluating the following surrogate markers with the reference line: (**a**) SPISE; (**b**) METS-IR, LAP, METS-VF, VAT, BRI, VAI, TG/HDL-C ratio, TyG, and ABSI.

**Figure 11:**
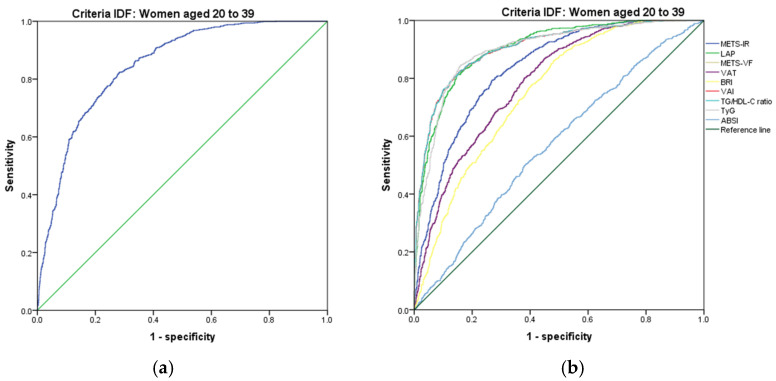
ROC curves for women aged 20–39 years according to the IDF criteria, evaluating the following surrogate markers with the reference line: (**a**) SPISE; (**b**) METS-IR, LAP, METS-VF, VAT, BRI, VAI, TG/HDL-C ratio, TyG, and ABSI.

**Figure 12:**
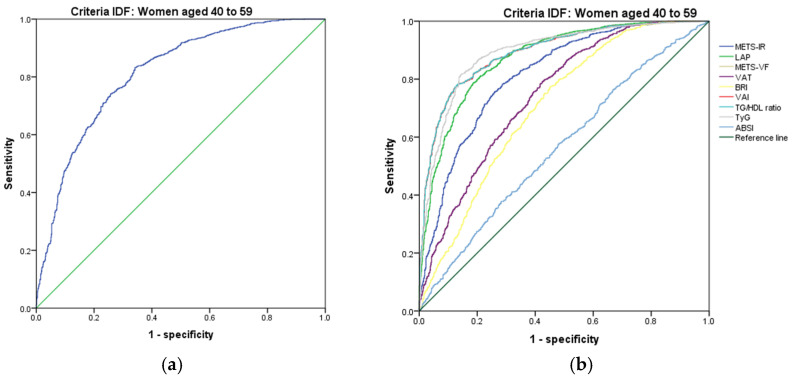
ROC curves for women aged 40–59 years according to the IDF criteria, evaluating the following surrogate markers with the reference line: (**a**) SPISE; (**b**) METS-IR, LAP, METS-VF, VAT, BRI, VAI, TG/HDL-C ratio, TyG, and ABSI.

Figure 7, Figure 8, Figure 9, Figure 10, Figure 11 and Figure 12 compare ROC curves, and Table 8, Table 9, Table 10, Table 11, Table 12 and Table 13 report the accuracy measures for the diagnostic of *MetS* according to the IDF criteria using the same markers as for the ATP-III criteria.

In men aged 20–39, we found that LAP had a higher Youden index (AUC = 0.902 (95% CI 0.888–0.916); Se = 90.3 (95% CI 88.0–92.4); Sp = 75.9 (95% CI 73.1–78.5), YI = 66.2 (95% CI 62.7–69.4) and cut-off value ≥ 57.97). The following markers had lower diagnostic accuracy than LAP: METS-IR (AUC = 0.895 (95% CI 0.881–0.910); Se = 89.5 (95% CI 87.1–91.6); Sp = 73.7 (95% CI 70.9–76.5); YI = 63.2 (95% CI 59.6–66.1); and cut-off value ≥ 43.29) and METS-VF (AUC = 0.859 (95% CI 0.841–0.876); Se = 86.0 (95% CI 83.3–88.4); Sp 70.8 (95% CI 67.8–73.6); YI = 56.8 (95% CI 53.1–60.6); and cut-off value ≥ 6.72). See Figure 8 and Table 8. We found no differences in the AUC for the diagnosis of *MetS* according to the IDF criteria between the LAP and METS-IR (*p* = 0.318). However, we found significant differences between METS-IR and METS-VF (*p* < 0.001). We chose LAP to diagnose *MetS* according to the IDF criteria in men aged 20–39. Although it has the same ability to discriminate as METS-IR, LAP only uses triglycerides and waist circumference.

In men aged 40–59, we found that METS-IR had a higher Youden index (AUC = 0.872 (95% CI 0.854–0.889); Se = 87.3 (95% CI 85.1–89.3); Sp = 69.5 (95% CI 65.9–73.0); YI = 56.8 (95% CI 52.6–60.8); and cut-off value ≥ 42.86). The following markers had lower diagnostic accuracy than METS-IR: LAP (AUC = 0.867 (95% CI 0.849–0.885); Se = 86.7 (95% CI 84.5–88.7); Sp = 69.6 (95% CI 66.0–73.1); YI = 56.3 (95% CI 52.2–60.1); and cut-off value ≥ 59.07) and SPISE (AUC = 0.863 (95% CI 0.845–0.881); Se = 65.4 (95% CI 62.4–68.3); Sp = 86.4 (95% CI 83.6–88.9); YI = 51.8 (95% CI 47.8–55.5); and cut-off value ≤ 4.44). See Figure 9 and Table 9. We found no differences in the AUC for the diagnosis of *MetS* according to the IDF criteria between the METS-IR and LAP (*p* = 0.558) and between LAP and SPISE (*p* = 0.543). As with the previous age group, we chose LAP to diagnose *MetS* according to the IDF criteria in men aged 40–59.

In men aged 60 and over, we found that METS-IR had a higher Youden index (AUC = 0.885 (95% CI 0.864–0.905); Se = 88.6 (95% CI 85.9–90.9); Sp = 71.5 (95% CI 67.0–75.6); YI = 60.1 (95% CI 55.2–64.9); and cut-off value ≥ 40.48). The following markers had lower diagnostic accuracy than METS-IR: LAP (AUC = 0.891 (95% CI 0.872–0.910); Se = 89.2 (95% CI 86.6–91.4); Sp = 70.4 (95% CI 65.9–74.6); YI = 59.6 (95% CI 54.8–64.4); and cut-off value ≥ 45.81) and METS-VF (AUC = 0.833 (95% CI 0.808–0.859); Se = 83.4 (95% CI 80.4–86.2); Sp = 69.2 (95% CI 64.7–73.5); YI = 52.6 (95% CI 47.5–57.9) and cut-off value ≥ 7.37). See Figure 10 and Table 10. We found no differences in the AUC for the diagnosis of *MetS* according to the IDF criteria between METS-IR and LAP (*p* = 0.479). However, we found significant differences between LAP and METS-VF (*p* = 0.116). As in the previous age group, we chose LAP to diagnose *MetS* according to the IDF criteria in men over 60.

In women aged 20–39, we found that TyG had a higher Youden index (AUC = 0.898 (95% CI 0.885–0.911); Se = 89.8 (95% CI 87.8–91.6); Sp = 73.3 (95% CI 70.8–75.7); YI = 63.1 (95% CI 60.1–66.1); and cut-off value ≥ 4.67). The following markers had lower diagnostic accuracy than TyG: LAP (AUC = 0.903 (95% CI 0.891–0.915); Se = 90.3 (95% CI 88.3–92.1); Sp = 71.1 (95% CI 68.6–73.6); YI = 61.4 (95% CI 58.2–64.4); and cut-off value ≥ 46.16) and VAI (AUC = 0.904 (95% CI 0.892–0.917); Se = 90.4 (95% CI 88.4–92.2); Sp = 70.0 (95% CI 67.4–72.6); YI = 60.4 (95% CI 57.3–63.4); and cut-off value ≥ 91.42). See Figure 11 and Table 11. We found no differences in the AUC for the diagnosis of *MetS* according to the IDF criteria between TyG and LAP (*p* = 0.308) and between LAP and VAI (*p* = 0.824). Although TyG has the same ability to discriminate as LAP, we chose TyG to diagnose *MetS* according to the IDF criteria in women aged 20–39.

In women aged 40–59, we found that TyG had a higher Youden index (AUC = 0.893 (95% CI 0.878–0.907); Se = 89.3 (95% CI 87.8–90.8); Sp = 74.7 (95% CI 71.4–77.8); YI = 64.0 (95% CI 60.5–67.4); and cut-off value ≥ 4.73). The following markers had lower diagnostic accuracy than TyG: LAP (AUC = 0.875 (95% CI 0.860–0.891); Se = 87.5 (95% CI 85.9–89.1); Sp = 70.1 (95% CI 66.6–73.4); YI = 57.6 (95% CI 53.9–61.2); and cut-off value ≥ 55.94) and VAI (AUC = 0.892 (95% CI 0.879–0.906); Se = 89.2 (95% CI 87.6–90.7); Sp = 68.0 (95% CI 64.5–71.4), YI = 57.2 (95% CI 53.5–60.9); and cut-off value ≥ 87.28). See Figure 12 and Table 12. We found significant differences in the AUC for the diagnosis of *MetS* according to the IDF criteria between TyG and LAP (*p* = 0.011) and between LAP and VAI (*p* = 0.013). We chose TyG to diagnose *MetS* according to the IDF criteria in women aged 40–59 because it has a greater discriminating capacity.

**Figure 13:**
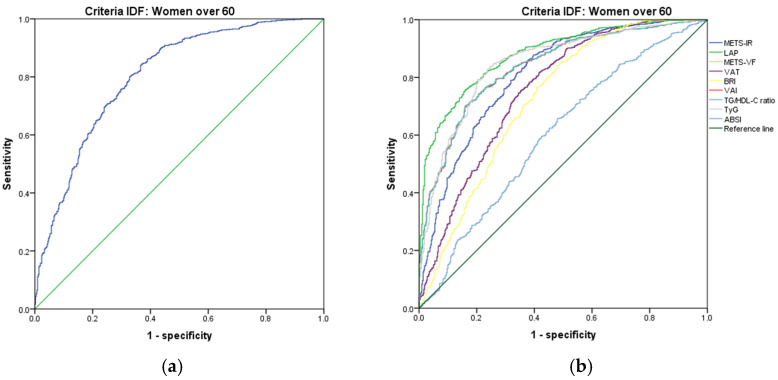
ROC curves for women over 60 years according to the IDF criteria, evaluating the following surrogate markers with the reference line: (**a**) SPISE; (**b**) METS-IR, LAP, METS-VF, VAT, BRI, VAI, TG/HDL-C ratio, TyG, and ABSI.

In women over 60 years, we found that TyG had a higher Youden index (AUC = 0.848 (95% CI 0.826–0.871); Se = 84.9 (95% CI 82.4–87.1); Sp = 73.6 (95% CI 69.1–77.7); YI = 58.5 (95% CI 53.8–63.2); and cut-off value ≥ 4.74). The following markers had lower diagnostic accuracy than TyG: LAP (AUC = 0.881 (95% CI 0.863–0.899); Se = 88.1 (95% CI 85.9–90.1); Sp = 66.0 (95% CI 61.2–70.5); YI = 54.1 (95% CI 49.1–59.0); and cut-off value ≥ 48.70) and TG/HDL ratio (AUC = 0.837 (95% CI 0.815–0.860); Se = 83.7 (95% CI 81.2–86.0); Sp = 66.9 (95% CI 62.2–71.4); YI = 50.6 (95% CI 45.5–55.0); and cut-off value ≥ 2.78). See Figure 13 and Table 13. We found significant differences in the AUC for the diagnosis of *MetS* according to the IDF criteria between TyG and LAP (*p* = 0.003) and between LAP and TG/HDL ratio (*p* < 0.001). We chose TyG to diagnose *MetS* according to the IDF criteria in women over 60 years old because it has a greater discriminating capacity.

## 4. Discussion

The present study investigated the value of SPISE, LAP, VAI, TyG, the TG/HDL ratio, METS-IR, METS -VF, and VAT, known and novel surrogate markers of insulin resistance and obesity, in identifying *MetS* according to different criteria in three age groups. Our results show that LAP and TyG have reliable predictive accuracy for diagnosing *MetS* in the ATP-III and IDF criteria. METS-IR had higher accuracy than LAP for diagnosing *MetS* according to the ATP criteria in men aged 20–39 and 40–59. Still, LAP could be used at the primary care level because it has acceptable accuracy. With the IDF criteria, LAP and TyG were better for diagnosing *MetS* in men and women. This study is the first report based on the analysis and comparison of four novel markers (METS-IR, METS-VF, VAT, and SPISE) for predicting *MetS* using different criteria in adult Mexicans in particular.

Several studies used obesity or insulin resistance markers to predict the *MetS* using the ATP-III criteria [15,20,23,33,34,35,36]. In an adult population in Taiwan, the following indices were used: BMI, WC, waist–hip ratio (WHR), WHtR, abdominal volume index (AVI), body roundness index (BRI), conicity index (CI), ABSI, body adiposity index (BAI), VAI, and TyG [15]. They found TyG and VAI to be the best predictors of *MetS* in clinical practice in both men (TyG: AUC = 0.850 (95% CI 0.835–0.864), Se = 79.4, Sp = 78.9, YI = 58.5, and cut-off value ≥ 8.83; VAI: AUC = 0.845 (95% CI 0.829–0.859); Se = 77.7, Sp = 77.2, YI = 54.9, and cut-off value ≥ 1.74) and women (TyG: AUC = 0.890 (95% CI 0.877–0.901), Se = 75.5, Sp = 88.6, YI = 64.1, and cut-off value ≥ 8.70; VAI: AUC = 0.888 (95% CI 0.876–0.900), Se = 77.6, Sp = 83.2, YI = 60.8 and cut-off value ≥ 1.83). They did not stratify by age. A small sample of postmenopausal Turkish women analyzed adiposity indicators such as VAI, LAP, and TyG [33] and found that VAI was the best predictor of *MetS* (AUC = 0.88 (95% CI 0.83–0.94); Se = 89.0; Sp = 80.0; YI = 69.0; and cut-off value ≥ 2.04). Our results in men show that the ability of VAI and TyG to discriminate by AUC and the Youden index is less than that reported by Chiu et al., except for the age group of 60 years and older, which show similar accuracy measures but with different cut-off values. For women, the age group from 20 to 39 years has measures of accuracy similar to Chiu et al.; for the other age groups, the discrimination of TyG and VAI is less in our results. The discriminant ability of VAI was better in the sample of Turkish women compared to our results in women aged 40–59 and 60 years and older.

In an Indian adult population, LAP, BMI, and WC were studied as predictors of *MetS* according to the ATP-III criteria modified for the Asian Population [20]. They reported that LAP was the best predictor (AUC = 0.901 (95% CI 0.85–0.95), Se = 76.4, Sp = 91.1, YI = 67.0, and cut-off value ≥ 38.05), but they did not stratify by age or sex. A study in middle-aged and elderly Chinese analyzed LAP, VAI, and TyG to predict *MetS* [34], and they found that LAP was the best predictor (AUC = 0.855 (95% CI 0.831–0.878), Se = 73.9, Sp = 79.7, YI = 53.6, and cut-off value ≥ 31.46). They also did not stratify by age or sex. A study of the Peruvian adult population examined BMI, LAP, VAI, and TyG for the diagnosis of *MetS* [35]. They reported that LAP was the best for diagnosing *MetS* in men (AUC = 0.929 (95% CI 0.907–0.952), Se = 91.6, Sp = 84.5, YI = 71.3, and cut-off value ≥ 59.85) and women (AUC = 0.950 (95% CI 0.940–0.960), Se = 92.4, Sp = 86.4, YI = 78.8, and cut-off value ≥ 53.06), and they did not stratify by age. In a population of Spanish adults, they analyzed different anthropometric indices (BMI, the Ponderal index, AVI, BAI, VAI, BRI, CI, Cholindex, WHR, and WHtR) and atherogenic indices (LAP; CT/HDL, LDL/HDL, and TG/HDL ratios; and non-HDL/HDL) [36]. They found that LAP was one of the best predictors of *MetS* in men (AUC = 0.946 (95% CI 0.943–0.950), Se = 96.0; Sp = 70.1, YI = 66.6, and cut-off value ≥ 18.40) and women (AUC = 0.942 (95% CI 0.935–0.950), Se = 95.0, Sp = 75.7, YI = 71.0, and cut-off value ≥ 36.04). Our results for the diagnostic capacity of LAP in men were not better than those reported in the populations of India, China, Peru, and Spain. By contrast, LAP in women had a better discriminative capacity than in the populations of India, China, and Spain but worse than in the population of Peru.

In the adult Mexican population, only the TG/HDL ratio (AUC = 0.853(95% CI 0.831–0.872), Se = 79.6, Sp = 76.4, YI = 55.95, and cut-off value ≥ 3.46) was studied to identify subjects with *MetS* in the Mexican population. Still, they did not stratify by sex or age [23]. In a population of Spanish adults [36], they also found that the TG/HDL ratio was one of the best predictors of *MetS* in men (AUC = 0.949 (95% CI 0.946–0.952), Se = 95.5, Sp = 81.8, YI = 77.7, and cut-off value ≥ 2.96) and women (AUC = 0.923 (95% CI 0.914–0.932), Se = 92.2, Sp = 70.6, YI = 63.0, and cut-off value ≥ 1.75). Our results showed that the discriminant capacity of the TG/HDL ratio using AUC and the Youden index was lower in men and higher in women, compared with those of Baez-Duarte et al. [36]. On the other hand, in the population of Spanish adults [36], the discriminant capacity of the TG/HDL ratio was better in men and women than in our study. However, the TG/HDL ratio could not be used at the primary care level, in contrast to LAP and TyG.

Studies using the ABSI index in the populations of Taiwan [15] and Spain [36] found very poor diagnostic performance, similar results to those found in men and women of different age groups in our study.

The ABSI in the studies consulted [15,36] has not been shown to be a good predictive indicator of *MetS*; similarly, in our study, despite the AUC of around 0.600, globally, it was the worst-performing indicator.

We have yet to find a study assessing the relationship between the METS-IR, SPISE, METS-VF, and VAT indices with *MetS* according to the ATP-III criteria. Therefore, we cannot make any comparisons with our results, which were acceptable to very good.

Several other studies analyzed the indices using the IDF criteria [34,37,38]. In a population of middle-aged and elderly Chinese [39], Li et al. studied LAP, VAI, and TyG, and they found that LAP (AUC = 0.865 (95% CI 0.841–0.889), Se = 73.2; Sp = 84.5, YI = 56.8, and cut-off value ≥ 37.99) was superior to VAI and TyG. They did not stratify by age or sex. In our results, LAP showed better discrimination capability in men and women aged 20–39 than in the study of Li et al.; in the other age groups, the results were similar. Our results on the diagnostic capacity of TyG in men and women of any age group showed that it was better than that in Li et al. (AUC = 0.746 (95% CI 0.712–0.779); Se = 70.2; Sp = 71.8, YI = 42.1; and cut-off value ≥ 8.697).

In a Chinese elderly population, they studied the BMI, WHtR, TG/HDL-C ratio, LAP, and VAI [39] and found that LAP was a better index in men (AUC = 0.897 (95% CI 0.885–0.907), Se = 85.09, Sp = 79.31, YI = 64.4, and cut-off value ≥ 26.35) and women (AUC = 0.875 (95% CI 0.864–0.886), Se = 79.17, Sp = 80.69, YI = 59.8; and cut-off value ≥ 31.4). LAP had a better diagnostic capacity in women and a similar one in men compared to the previous study. Our results on the diagnostic capacity of the TG/HDL index in women of any age group and men aged 20–39 showed that it was better than that in Gu. et al. (men; AUC = 0.851 (95% CI 0.838–0.864), Se = 69.47, Sp = 86.72, YI = 56.19, and cut-off value ≥ 1.38; women: AUC = 0.843 (95% CI 0.831–0.855), Se = 71.09, Sp = 82.76, YI = 53.85, and cut-off value ≥ 1.13), but it was very similar in men over 40 years.

In an adult population of Iranis analyzed BMI, ABSI, BRI, and VAI [38], it was reported that VAI was the best index in men (AUC = 0.824 (95% CI 0.812–0.836), Se = 80.08, Sp = 70.0, YI = 51.20, and cut-off value ≥ 4.12) and women (AUC = 0.866 (95% CI 0.855–0.877), Se = 83.1, Sp = 70.0, YI = 58.00, and cut-off value ≥ 4.28). In this work, they did not analyze LAP and TyG. Our results for men and women aged 20–39 years showed that VAI had a better diagnostic capacity than the results of Baveicy et al.; for men and women over 40 years, the discrimination capacity of VAI was similar. Our results, as well as those of Baveicy et al., showed that ABSI performed poorly in *MetS* diagnosis.

Insulin resistance and obesity markers for diagnosing *MetS* have been analyzed according to other criteria, such as the American Heart Association/National Heart Lung and Blood Institute (AHA/NHLBI) and the Harmonized International Diabetes Federation (HIDF). In a Chinese adult population, they analyzed TyG, METS-IR, and TG/HDL ratio indexes [39], and they found that TyG was the best one for diagnosing *MetS* according to the HIDF criteria in men (AUC = 0.863 (95% CI 0.857–0.869); Se = 77.47; Sp = 83.55; YI = 0.585; and cut-off value ≥ 8.81) and women (AUC = 0.867 (95% CI 0.862–0.872); Se = 71.49; Sp = 88.57; YI = 60.06; and cut-off value ≥ 8.73). In the middle-aged and older Korean population, they used markers of body fat distribution, such as LAP, VAI, TyG, and WHtR to diagnose *MetS* according to the AHA/NHLBI criteria [19]. They found that LAP was the best for men (AUC = 0.899 (95% CI 0.893–0.906), Se = 83.6; Sp = 82.8, YI = 66.4, and cut-off value ≥ 40.78) and women (AUC = 0.953 (95% CI 0.946–0.960), Se = 92.6, Sp = 85.5, YI = 78.1, and cut-off value ≥ 23.85). In previous studies, it was also documented that LAP and TyG are good for diagnosing *MetS*.

LAP and TyG have shown good accuracy in various populations but have also shown variability in the selection of cut-off values for the diagnosis of *MetS*, even when the same criteria are used. As we have been able to review, in most LAP and TyG studies, they are useful for predicting *MetS*. Considering that these indices are easy, fast to calculate, and inexpensive, they could be an alternative to MetS screening at the first level of attention, even more so in countries where access to certain biochemical tests is limited.

The LAP is a marker that combines measures of abdominal obesity and triglyceride levels to provide insights into lipid overaccumulation and metabolic dysfunction [26]. This is explained by the fact that visceral fat has a high lipolytic capacity, so it is able to release fatty acids into the circulation, which in turn can be taken up by the liver, which uses them to form glucose. This means that the greater the waist circumference, the higher the triglyceridemia, hyperglycemia, and insulin resistance. TyG is an indicator of insulin resistance because when you have a high concentration of glucose, insulin secretion is stimulated, which is a physiological mechanism, but if this hyperglycemia is sustained, the pancreas loses the ability to produce insulin in the necessary amounts [40], which makes it especially useful in the diagnosis of diabetes mellitus.

Lipid metabolism is influenced by the circadian clock, which is modulated by light exposure and 24-h dietary patterns. Research on circadian rhythms of triglycerides consistently reveals a peak during the night [41], often aligning closely with the melatonin phase, although the strength of this rhythm varies among individuals [41]. However, nocturnal eating habits can alter nocturnal triglyceride levels, potentially shifting toward a daytime pattern similar to that of HDL and LDL cholesterol, which are less affected by nocturnal meals [42]. Additionally, administering melatonin before the evening meal may enhance postprandial triglyceride levels [43].

Triglycerides’ sensitivity as a biomarker can be attributed to circadian factors (note that metabolic syndrome can even be considered circadian syndrome [44,45]). Nocturnal eating habits and compromised metabolic health are closely associated with an evening chronotype [46] and exposure to evening light [47], which may also contribute to the development of metabolic syndrome [48]. Furthermore, lipid measurements such as triglycerides or HDL sampled at a fixed morning time may vary depending on individual circadian phase differences, such as melatonin secretion or light exposure (e.g., [49]). This variability can influence the interpretation of results obtained from a single morning sample. Conversely, it suggests that indicators of metabolic syndrome are intricately linked with disrupted circadian rhythms, evident in delayed light exposure and/or meal timing.

One of the strengths of our study is that a sample with national representation was obtained from ENSANUT-2018. However, this study has several limitations. The cross-sectional design cannot evaluate longitudinal relationships between these surrogate insulin resistance and obesity markers and *MetS*. The participants in our study were limited to Mexicans; therefore, our results might not be generalizable to other populations.

## 5. Conclusions

According to the ATP III criteria for diagnosing *MetS* in Mexican adult men aged 20–39 and 40–59 years, the best biomarker according to the Youden index and AUC was METS-IR, but LAP could be used at the primary care level because it has acceptable accuracy. Conversely, for participants ≥60 years old, the best marker was LAP. Nonetheless, the best biomarker in women aged 20–39 years was LAP; however, for 40–59 and ≥60-year-old women, it was TyG. On the other hand, using the IDF criteria for men aged 20–39, 40–59, and ≥60 years old, the best marker was LAP. In women aged 20–39, 40–59, and ≥60 years old, the best marker was TyG.

LAP and TyG were shown to be reliable markers for the diagnosis of *MetS* in adults. In addition, by requiring only the levels of serum triglycerides and glucose to calculate TyG, or the waist circumference and serum triglycerides to determine LAP, it is simple to calculate, accessible to request in patients’ laboratories by professionals, and inexpensive for primary care. All of the above findings give doctors a more precise and accurate view of patients’ cardiometabolic health, allowing for the early diagnosis and treatment of *MetS*. LAP and TyG must be included in our lipid profile reports.

## Figures and Tables

**Table 1:** Sociodemographic, anthropometric, and biochemical characteristics of 10575 Mexican adults with suspected metabolic syndrome according to ENSANUT 2018.

Variables		*n* (%)
Female		6041 (57%)
Age, median (Q1, Q3)	years	45 (33, 58)
Age groups	20–39 years	4006 (37.9%)
	40–59 years	4095 (38.7%)
	≥60 years	2474 (23.4%)
BMI, median (Q1, Q3)	kg/m^2^	28.4 (25.2, 31.9)
	≤18.4 kg/m^2^	106 (1.0%)
	18.5–24.4 kg/m^2^	2288 (21.6%)
	25–29.9 kg/m^2^	2606 (24.6%)
	≥30 kg/m^2^	5575 (52.7%)
Waist circumference, median (Q1, Q3)	cm	95.8 (87.5, 104)
	ATP-III Men WC ≥ 102 cm ATP-III Woman WC ≥ 88 cm IDF Men WC ≥ 90 cm	1679 (37.0%) 4445 (73.6%) 3296 (72.7%)
	IDF Woman WC ≥ 80 cm	5369 (88.9%)
Systolic pressure, median (Q1, Q3)	mmHg	122 (111, 135)
	≥135 mmHg	3661 (34.6%)
Diastolic pressure, median (Q1,Q3)	mmHg	75 (68, 82)
	≥85 mmHg	2185 (20.7%)
Cholesterol, median (Q1,Q3)	mg/dL	183 (159, 209)
	≥200 mg/dL	3555 (33.6%)
High-density-lipoprotein cholesterol, median (Q1,Q3)	mg/dL Men HDL-C ≤ 40 mg/dL Women HDL-C ≤ 50 mg/dL	43 (37, 50) 4325 (40.9%) 8091 (76.5%)
Triglycerides, median (Q1,Q3)	mg/dL	168 (117, 243)
	≥150 mg/dL	6201 (58.6%)
Glucose, median (Q1,Q3)	mg/dL	92 (84, 103)
	ATP III Glu ≥ 110 mg/dL	2025 (19.1%)
	IDF Glu ≥ 100 mg/dL	3216 (30.4%)
*MetS* ATP III	Men	1808 (39.9%)
	Woman	3180 (52.6%)
*MetS* IDF	Men	2431 (53.6%)
	Woman	3624 (60.0%)

Q1: Quartile 1; Q3: Quartile 3; ATP III: Adult Treatment Panel III of the National Cholesterol Education Program; IDF: International Diabetes Federation; *MetS*: metabolic syndrome.

**Table 2:** Accuracy measures of surrogate insulin resistance and obesity markers to identify metabolic syndrome in Mexican adult men aged 20–39 according to the ATP III criteria. The sample included 506 subjects with *MetS* and 1216 without *MetS*.

Index	Cut-Off Point	AUC (95% CI)	Se (%)	Sp (%)	PPV (%)	NPV (%)	LRP	LRN	Youden Index
METS-IR	≥45.29	0.884	88.5	71.3	56.2	93.7	3.08	0.16	59.8
		(0.868–0.900)	(85.4 –91.1)	(68.6–73.8)	(53.8–58.5)	(92.1–95.0)	(2.81–3.39)	(0.13–0.21)	(56.0–63.6)
SPISE	≤4.13	0.875	67.8	87.5	69.7	86.8	5.53	0.37	55.3
		(0.858–0.892)	(63.5–71.8)	(85.8–89.5)	(66.2–73.2)	(85.3–88.3)	(4.70–6.50)	(0.32–0.42)	(51.0–59.9)
LAP	≥65.22	0.855	85.5	69.1	53.5	92.0	2.77	0.21	54.6
		(0.837–0.873)	(82.2–88.5)	(66.4–71.7)	(51.3–55.8)	(90.2–93.4)	(2.53–3.04)	(0.17–0.26)	(50.6–58.6)
BRI	≥4.70	0.836	83.7	69.5	53.4	91.1	2.75	0.23	53.2
		(0.815–0.856)	(80.2–86.9)	(66.9–72.1)	(51.0–55.7)	(89.4–92.6)	(2.51–3.02)	(0.19–0.28)	(49.1–0.573)
METS-VF	≥6.79	0.849	84.9	67.2	51.9	91.5	2.6	0.22	52.1
		(0.829–0.868)	(81.5–87.9)	(64.5–69.9)	(49.7–54.1)	(89.7–93.0)	(2.38–2.84)	(0.18–0.28)	(48.0–56.1)
VAT	≥895.52	0.849	84.9	67.2	51.9	91.5	2.6	0.22	52.1
		(0.829–0.868)	(81.5–87.9)	(64.5–69.9)	(49.7–54.1)	(89.7–93.0)	(2.38–2.84)	(0.18–0.28)	(48.0–56.1)
TG/HDL	≥4.59	0.803	80.4	67.1	50.4	89.1	2.45	0.29	47.5
		(0.782–0.824)	(76.7–83.8)	(64.3–69.7)	(48.1–52.7)	(87.3–90.8)	(2.23–2.68)	(0.24–0.35)	(43.1–51.8)
VAI	≥105.75	0.803	80.4	67.1	50.4	89.1	2.45	0.29	47.5
		(0.781–0.824)	(76.7–83.8)	(64.3–69.7)	(48.1–52.7)	(87.3–90.8)	(2.23–2.68)	(0.24–0.35)	(43.1–51.8)
TyG	≥4.87	0.785	78.6	65.3	48.5	88	2.27	0.33	43.9
		(0.763–0.807)	(74.8–82.1)	(62.5–67.9)	(46.3–50.7)	(86.0–89.7)	(2.07–2.48)	(0.28–0.39)	(39.4–48.4)
ABSI	≥0.07	0.614	62	54.2	36	77.4	1.36	0.7	16.2
		(0.585–0.642)	(57.6–66.3)	(51.4–57.1)	(34.0–38.2)	(75.2–79.5)	(1.24–1.49)	(0.62–0.79)	(11.0–21.2)

ATP III: Adult Treatment Panel III of the National Cholesterol Education Program; AUC: area under the curve; Se: sensitivity; Sp: specificity; PPV: positive predictive value; NPV: negative predictive value; LRP: positive likelihood ratio; LRN: negative likelihood ratio; SPISE: single-point insulin sensitivity estimator; TG/HDL: triglyceride–HDL ratio; TyG: triglyceride–glucose index; LAP: lipid accumulation product; VAI: visceral adiposity index; METS-IR: metabolic score for insulin resistance; METS-VF: metabolic score for visceral fat; VAT: visceral adipose tissue; ABSI: body shape index; BRI: body rounding index.

**Table 3:** Accuracy measures of surrogate insulin resistance and obesity markers for the identification of metabolic syndrome in Mexican adult men aged 40–59 according to the ATP III criteria. The sample included 766 subjects with *MetS* and 941 without *MetS*.

Index	Cut-Off Point	AUC (95% CI)	Se (%)	Sp (%)	PPV (%)	NPV (%)	LRP	LRN	Youden Index
METS-IR	≥45.08	0.867	86.8	68.0	68.8	86.3	2.71	0.19	54.8
		(0.850–0.883)	(84.2–89.1)	(64.9–70.9)	(66.7–70.8)	(84.0–88.4)	(2.46–2.99)	(0.16–0.23)	(50.9–58.6)
LAP	≥70.75	0.836	83.6	68.8	68.6	83.8	2.69	0.24	52.4
		(0.817–0.854)	(80.8–86.2)	(65.8–71.8)	(66.4–70.7)	(81.4–85.9)	(2.43–2.97)	(0.20–0.28)	(48.3–56.3)
SPISE	≤4.22	0.846	64.1	84.7	77.3	74.3	4.19	0.42	48.8
		(0.828–0.865)	(60.5–67.5)	(82.2–86.9)	(74.4–80.0)	(72.4–76.1)	(3.57–4.91)	(0.38–0.47)	(44.7–52.7)
METS-VF	≥7.21	0.826	82.7	65.0	65.8	82.2	2.37	0.26	47.7
		(0.806–0.845)	(79.9–85.3)	(61.8–68.0)	(63.7–67.8)	(79.7–84.5)	(2.16–2.60)	(0.23–0.31)	(43.5–51.7)
VAT	≥1357.18	0.826	82.7	65	65.8	82.2	2.37	0.26	47.7
		(0.806–0.845)	(79.9–85.3)	(61.8–68.0)	(63.7–67.8)	(79.7–84.5)	(2.16–2.60)	(0.23–0.31)	(43.5–51.7)
TG/HDL	≥4.48	0.796	79.7	64.8	64.8	79.7	2.27	0.31	44.5
		(0.776–0.817)	(76.7–82.5)	(61.6–67.8)	(62.7–66.9)	(77.2–82.0)	(2.06–2.49)	(0.27–0.36)	(40.3–48.7)
VAI	≥103.21	0.796	79.7	64.5	64.6	79.6	2.25	0.31	44.2
		(0.775–0.817)	(76.7–82.5)	(61.3–67.5)	(62.5–66.7)	(77.1–81.9)	(2.05–2.47)	(0.27–0.36)	(40.0–48.4)
TyG	≥4.90	0.792	79.3	64.4	64.4	79.3	2.23	0.32	43.7
		(0.771–0.813)	(76.3–82.1)	(61.2–67.4)	(62.3–66.5)	(76.8–81.6)	(2.03–2.45)	(0.28–0.37)	(39.4–47.7)
BRI	≥5.17	0.788	78.8	63.3	63.6	78.6	2.15	0.33	42.1
		(0.767–0.810)	(75.7–81.6)	(60.1–66.4)	(61.5–65.7)	(76.0–80.9)	(1.96–2.36)	(0.29–0.39)	(37.9–46.3)
ABSI	≥0.08	0.609	61.7	54	52.2	63.4	1.35	0.71	15.7
		(0.582–0.635)	(58.2–65.2)	(50.8–57.3)	(50.0–54.4)	(60.9–65.9)	(1.23–1.47)	(0.64–0.79)	(11.2–20.3)

ATP III: Adult Treatment Panel III of the National Cholesterol Education Program; AUC: area under the curve; Se: sensitivity; Sp: specificity; PPV: positive predictive value; NPV: negative predictive value; LRP: positive likelihood ratio; LRN: negative likelihood ratio; SPISE: single-point insulin sensitivity estimator; TG/HDL: triglyceride–HDL ratio; TyG: triglyceride–glucose index; LAP: lipid accumulation product; VAI: visceral adiposity index; METS-IR: metabolic score for insulin resistance; METS-VF: metabolic score for visceral fat; VAT: visceral adipose tissue; ABSI: body shape index; BRI: body rounding index.

**Table 4:** Accuracy measures of surrogate insulin resistance and obesity markers to identify metabolic syndrome in Mexican adult men over 60 according to the ATP III criteria. The sample included 536 subjects with *MetS* and 569 without *MetS*.

Index	Cut-Off Point	AUC (95% CI)	Se (%)	Sp (%)	PPV (%)	NPV (%)	LRP	LRN	Youden Index
LAP	≥55.83	0.876	87.6	73.2	75.5	86.3	3.28	0.17	60.8
		(0.855–0.896)	(84.6–90.3)	(69.4–76.8)	(72.8–78.0)	(83.3–88.8)	(2.85–3.77)	(0.13–0.21)	(56.8–65.8)
TG/HDL	≥3.84	0.862	86.3	72.9	75	85	3.19	0.19	59.2
		(0.840–0.883)	(83.1–89.1)	(69.0–76.5)	(72.3–77.5)	(82.0–87.6)	(2.78–3.67)	(0.15–0.23)	(54.7–64.3)
VAI	≥89.26	0.861	86.1	72.9	75	84.8	3.18	0.19	59.0
		(0.839–0.883)	(82.9–89.0)	(69.0–76.5)	(72.3–77.5)	(81.8–87.4)	(2.77–3.66)	(0.15–0.24)	(54.1–63.6)
TyG	≥4.80	0.858	86.0	71.8	74.2	84.5	3.06	0.19	57.8
		(0.836–0.880)	(82.7–88.8)	(67.9–75.5)	(71.5–76.7)	(81.4–87.1)	(2.67–3.50)	(0.16–0.24)	(53.1–62.5)
METS-IR	≥41.91	0.88	88.0	69.7	73.2	86.1	2.91	0.17	57.7
		(0.860–0.900)	(85.0–90.6)	(65.8–73.5)	(70.7–75.7)	(83.0–88.7)	(2.56–3.31)	(0.14–0.22)	(53.2–62.4)
SPISE	≤4.75	0.867	67.9	86.8	82.9	74.1	5.15	0.37	54.7
		(0.846–0.888)	(63.7–71.8)	(83.7–89.4)	(79.5–85.8)	(71.6–76.5)	(4.14–6.41)	(0.33–0.42)	(50.0–59.6)
METS-VF	≥7.43	0.798	79.8	63.9	67.6	77.1	2.22	0.31	43.7
		(0.772–0.824)	(76.2–83.1)	(59.8–67.9)	(64.9–70.1)	(73.8–80.1)	(1.97–2.49)	(0.26–0.38)	(38.6–49.0)
VAT	≥1693.07	0.798	79.8	63.9	67.6	77.1	2.22	0.31	43.7
		(0.772–0.824)	(76.2–83.1)	(59.8–67.9)	(64.9–70.1)	(73.8–80.1)	(1.97–2.49)	(0.26–0.38)	(38.6–49.0)
BRI	≥0.08	0.763	76.4	62.9	66.0	73.9	2.06	0.37	39.3
		(0.736–0.791)	(72.6–80.0)	(58.8–66.9)	(63.3–68.5)	(70.6–77.0)	(1.84–2.32)	(0.32–0.44)	(34.0–44.7)
ABSI	≥5.31	0.575	58.9	52.9	54.1	57.7	1.25	0.78	11.8
		(0.541–0.608)	(54.6–63.1)	(48.7–57.0)	(51.3–56.8)	(54.6–60.8)	(1.12–1.40)	(0.68–0.88)	(6.1–17.8)

ATP III: Adult Treatment Panel III of the National Cholesterol Education Program; AUC: area under the curve; Se: sensitivity; Sp: specificity, PPV: positive predictive value; NPV: negative predictive value; LRP: positive likelihood ratio; LRN: negative likelihood ratio; SPISE: single-point insulin sensitivity estimator; TG/HDL: triglyceride–HDL ratio; TyG: triglyceride–glucose index; LAP: lipid accumulation product; VAI: visceral adiposity index; METS-IR: metabolic score for insulin resistance; METS-VF: metabolic score for visceral fat; VAT: visceral adipose tissue; ABSI: body shape index; BRI: body rounding index.

**Table 5:** Accuracy measures of surrogate markers of insulin resistance and obesity to identify metabolic syndrome in Mexican adult women aged 20–39 according to the ATP III criteria. The sample consisted of 825 subjects with *MetS* and 1459 without *MetS*.

Index	Cut-Off Point	AUC (95% CI)	Se (%)	Sp (%)	PPV (%)	NPV (%)	LRP	LRN	Youden Index
LAP	≥56.01	0.924	92.4	78.1	70.5	94.8	4.23	0.1	70.5
		(0.913–0.935)	(90.4–94.1)	(75.9–80.2)	(68.4–72.5)	(93.5–95.9)	(3.83–4.67)	(0.08–0.12)	(67.6–73.2)
TyG	≥4.73	0.88	88.1	77.1	68.5	91.9	3.86	0.15	65.2
		(0.866–0.895)	(85.7–90.2)	(74.9–79.3)	(66.4–70.6)	(90.5–93.2)	(3.50–4.26)	(0.13–0.19)	(62.0–68.2)
TG/HDL	≥3.31	0.888	88.9	74.9	66.7	92.3	3.55	0.15	63.8
		(0.874–0.901)	(86.6–91.0)	(72.6–77.1)	(64.6–68.7)	(90.8–93.5)	(3.24–3.89)	(0.12–0.18)	(60.6–66.8)
VAI	≥104.08	0.887	88.8	74.7	66.5	92.2	3.52	0.15	63.5
		(0.874–0.901)	(86.5–90.9)	(72.4–76.9)	(64.5–68.5)	(90.7–93.5)	(3.21–3.86)	(0.12–0.18)	(60.4–66.6)
METS-IR	≥43.91	0.869	87.0	72.1	63.8	90.7	3.13	0.18	59.1
		(0.855–0.884)	(84.5–89.2)	(69.8–74.4)	(61.8–65.8)	(89.1–92.1)	(2.87–3.41)	(0.15–0.22)	(55.9–62.2)
SPISE	≤4.37	0.882	69.7	88.2	77.0	83.7	5.91	0.34	57.9
		(0.868–0.896)	(66.4–72.8)	(86.5–89.8)	(74.3–79.5)	(82.2–85.1)	(5.10–6.85)	(0.31–0.38)	(54.3–61.4)
METS-VF	≥7.23	0.836	83.7	67.3	59.1	87.9	2.56	0.24	51.0
		(0.820–0.852)	(81.0–86.2)	(64.8–69.7)	(57.2–61.0)	(86.2–89.5)	(2.37–2.77)	(0.21–0.28)	(47.5–54.5)
VAT	≥1388.44	0.836	83.7	67.3	59.1	87.9	2.56	0.24	51.0
		(0.820–0.852)	(81.0–86.2)	(64.8–69.7)	(57.2–61.0)	(86.2–89.5)	(2.37–2.77)	(0.21–0.28)	(47.5–54.5)
BRI	≥5.21	0.808	80.9	67.0	58.1	86.1	2.46	0.28	47.9
		(0.791–0.825)	(78.1–83.5)	(64.5–69.4)	(56.1–60.0)	(84.3–87.8)	(2.27–2.66)	(0.25–0.33)	(44.3–0.513)
ABSI	≥0.07	0.597	60.4	54.0	42.6	70.7	1.32	0.73	14.4
		(0.573–0.621)	(57.0–63.8)	(54.4–56.6)	(40.7–44.6)	(68.7–72.7)	(1.22–1.42)	(0.66–0.80)	(10.1–18.6)

ATP III: Adult Treatment Panel III of the National Cholesterol Education Program; AUC: area under the curve; Se: sensitivity; Sp: specificity; PPV: positive predictive value; NPV: negative predictive value; LRP: positive likelihood ratio; LRN: negative likelihood ratio; SPISE: single-point insulin sensitivity estimator; TG/HDL: triglyceride–HDL ratio; TyG: triglyceride–glucose index; LAP: lipid accumulation product; VAI: visceral adiposity index; METS-IR: metabolic score for insulin resistance; METS-VF: metabolic score for visceral fat; VAT: visceral adipose tissue; ABSI: body shape index; BRI: body rounding index.

**Table 6:** Accuracy measures of surrogate insulin resistance and obesity markers to identify metabolic syndrome in Mexican adult women aged 40–59 according to the ATP III criteria. The sample included 1471 subjects with *MetS* and 917 without *MetS*.

Index	Cut-Off Point	AUC (95% CI)	Se (%)	Sp (%)	PPV (%)	NPV (%)	LRP	LRN	Youden Index
TyG	≥4.77	0.866	86.6	71.7	83.1	77	3.07	0.19	58.3
		(0.851–0.881)	(84.8–88.3)	(68.7–74.6)	(81.5–84.5)	(74.5–79.3)	(2.76–3.41)	(0.16–0.21)	(54.9–61.6)
TG/HDL	≥3.16	0.866	86.7	71.4	82.9	77.0	3.04	0.19	58.1
		(0.850–0.881)	(84.9–88.4)	(68.3–74.3)	(81.4–84.3)	(74.5–79.3)	(2.74–3.37)	(0.16–0.21)	(54.6–61.5)
LAP	≥60.23	0.881	88.1	69.9	82.4	78.6	2.93	0.17	58.0
		(0.867–0.895)	(86.4–89.7)	(66.8–72.8)	(80.9–83.8)	(76.1–81.0)	(2.65–3.24)	(0.15–0.20)	(54.6–61.3)
VAI	≥99.27	0.865	86.6	71.3	82.8	76.8	3.02	0.19	57.9
		(0.850–0.881)	(84.7–88.3)	(68.2–74.2)	(81.3–84.3)	(74.3–79.1)	(2.72–3.35)	(0.16–0.22)	(54.5–61.2)
METS-IR	≥44.69	0.832	83.2	68.4	80.9	71.8	2.64	0.24	51.6
		(0.815–0.849)	(81.2–85.1)	(65.3–71.4)	(79.3–82.3)	(69.3–74.2)	(2.40–2.91)	(0.22–0.28)	(47.9–55.0)
SPISE	≤4.34	0.826	63.6	82.6	85.4	58.6	3.65	0.44	46.2
		(0.809–0.843)	(61.1–66.0)	(80.0–85.0)	(83.5–87.2)	(56.8–60.4)	(3.15–4.22)	(0.41–0.47)	(42.7–49.6)
METS-VF	≥7.62	0.779	78.0	64.3	77.8	64.6	2.19	0.34	42.3
		(0.759–0.799)	(75.8–80.1)	(61.1–67.4)	(76.2–79.3)	(62.1–67.0)	(2.00–2.40)	(0.31–0.38)	(38.6–45.9)
VAT	≥2044.70	0.779	77.9	64.3	77.8	64.5	2.19	0.34	42.2
		(0.759–0.799)	(75.7–80.0)	(61.1–67.4)	(76.2–79.3)	(62.0–66.9)	(2.00–2.40)	(0.31–0.38)	(38.5–45.9)
BRI	≥5.77	0.744	74.5	63.2	76.4	60.7	2.03	0.4	37.7
		(0.723–0.766)	(72.2–76.7)	(60.0–66.3)	(74.8–78.0)	(58.3–63.1)	(1.85–2.22)	(0.36–0.45)	(72.3–76.7)
ABSI	≥0.08	0.585	59.1	52.5	66.6	44.5	1.25	0.78	11.6
		(0.561–0.608)	(56.5–61.6)	(49.2–55.8)	(64.8–68.4)	(42.3–46.6)	(1.15–1.35)	(0.71–0.85)	(7.5–15.6)

ATP III: Adult Treatment Panel III of the National Cholesterol Education Program; AUC: area under the curve; Se: sensitivity; Sp: specificity; PPV: positive predictive value; NPV: negative predictive value; LRP: positive likelihood ratio; LRN: negative likelihood ratio; SPISE: single-point insulin sensitivity estimator; TG/HDL: triglyceride–HDL ratio; TyG: triglyceride–glucose index; LAP: lipid accumulation product; VAI: visceral adiposity index; METS-IR: metabolic score for insulin resistance; METS-VF: metabolic score for visceral fat; VAT: visceral adipose tissue; ABSI: body shape index; BRI: body rounding index.

**Table 7:** Accuracy measures of surrogate insulin resistance and obesity markers to identify metabolic syndrome in Mexican adult women over 60 according to ATP III criteria. The sample consisted of 884 subjects with *MetS* and 485 without *MetS*.

Index	Cut-Off Point	AUC (95% CI)	Se (%)	Sp (%)	PPV (%)	NPV (%)	LRP	LRN	Youden Index
TyG	≥4.75	0.86	86.0	74.0	85.7	74.4	3.31	0.19	60.0
		(0.839–0.880)	(83.6–88.3)	(69.8–77.8)	(83.8–87.5)	(71.0–77.6)	(2.84–3.86)	(0.16–0.22)	(55.3–64.5)
TG/HDL	≥2.84	0.857	85.8	68.2	83.1	72.5	2.7	0.21	54.0
		(0.836–0.877)	(83.3–88.0)	(63.9–72.3)	(81.1–84.9)	(69.0–75.9)	(2.37–3.09)	(0.17–0.25)	(49.2–58.6)
VAI	≥89.27	0.856	85.7	68.0	83.0	72.3	2.68	0.21	53.7
		(0.836–0.877)	(83.2–87.9)	(63.6–72.1)	(81.0–84.8)	(68.7–75.6)	(2.35–3.06)	(0.18–0.25)	(49.0–58.4)
LAP	≥51.79	0.871	87.2	65.9	82.3	73.9	2.56	0.19	53.1
		(0.853–0.890)	(84.8–89.3)	(61.5–70.1)	(80.4–84.1)	(70.2–77.2)	(2.26–2.91)	(0.16–0.23)	(48.3–57.9)
METS-IR	≥41.35	0.808	80.8	65.7	81.1	65.3	2.36	0.29	46.5
		(0.783–0.833)	(78.1–83.4)	(61.3–69.9)	(79.1–83.0)	(61.9–68.6)	(2.08–2.68)	(0.25–0.34)	(41.5–51.5)
SPISE	≤ 4.75	0.8	62.4	80.2	85.1	53.9	3.15	0.47	42.6
		(0.775–0.826)	(59.1–65.6)	(76.3–83.6)	(82.6–87.3)	(51.5–56.3)	(2.62–3.80)	(0.43–0.52)	(37.8–47.3)
METS-VF	≥7.86	0.747	74.8	65.3	79.7	58.8	2.16	0.38	40.1
		(0.719–0.776)	(71.8–77.7)	(60.9–69.5)	(77.6–81.7)	(55.6–61.9)	(1.90–2.46)	(0.34–0.44)	(35.0–45.2)
VAT	≥2611.89	0.747	74.7	65.3	79.7	58.7	2.61	0.39	40.0
		(0.719–0.776)	(71.7–77.6)	(60.9–69.5)	(77.5–81.7)	(55.5–61.8)	(1.90–2.45)	(0.34–0.44)	(34.9–45.1)
BRI	≥6.10	0.718	71.9	64.1	78.5	55.6	2.01	0.44	36.0
		(0.688–0.748)	(68.8–74.8)	(59.6–68.4)	(76.3–80.5)	(52.5–58.6)	(1.77–2.27)	(0.39–0.50)	(30.9–41.2)
ABSI	≥0.08	0.596	60.2	54.6	70.7	43.0	1.33	0.73	14.8
		(0.564–0.627)	(56.9–63.5)	(50.0–59.1)	(68.4–73.0)	(40.2–45.8)	(1.19–1.49)	(0.65–0.82)	(9.3–20.3)

ATP III: Adult Treatment Panel III of the National Cholesterol Education Program; AUC: area under the curve; Se: sensitivity; Sp: specificity; PPV: positive predictive value; NPV: negative predictive value; LRP: positive likelihood ratio; LRN: negative likelihood ratio; SPISE: single-point insulin sensitivity estimator; TG/HDL: triglyceride–HDL ratio; TyG: triglyceride–glucose index; LAP: lipid accumulation product; VAI: visceral adiposity index; METS-IR: metabolic score for insulin resistance; METS-VF: metabolic score for visceral fat; VAT: visceral adipose tissue; ABSI: body shape index; BRI: body rounding index.

**Table 8:** Accuracy measures of surrogate insulin resistance and obesity markers to identify metabolic syndrome in Mexican adult men aged 20–39 according to the IDF criteria. The sample consisted of 738 subjects with *MetS* and 984 without *MetS*.

Index	Cut-Off Point	AUC (95% CI)	Se (%)	Sp (%)	PPV (%)	NPV (%)	LRP	LRN	Youden Index
LAP	≥57.97	0.902	90.3	75.9	73.7	91.3	3.75	0.13	66.2
		(0.888–0.916)	(88.0–92.4)	(73.1–78.5)	(71.5–75.9)	(89.3–92.9)	(3.35–4.20)	(0.10–0.16)	(62.7–69.4)
METS-IR	≥43.29	0.895	89.5	73.7	71.9	90.4	3.42	0.14	63.2
		(0.881–0.910)	(87.1–91.6)	(70.9–76.5)	(69.7–74.0)	(88.3–92.1)	(3.07–3.80)	(0.11–0.18)	(59.6–66.1)
METS-VF	≥6.72	0.859	86.0	70.8	68.8	87.1	2.95	0.2	56.8
		(0.841–0.876)	(83.3–88.4)	(67.8–73.6)	(66.6–71.0)	(84.9–89.0)	(2.67–3.27)	(0.16–0.24)	(53.1–60.6)
VAT	≥831.33	0.859	86.0	70.8	68.8	87.1	2.95	0.2	56.8
		(0.841–0.876)	(83.3–88.4)	(67.8–73.6)	(66.6–71.0)	(84.9–89.0)	(2.67–3.27)	(0.16–0.24)	(53.1–60.6)
TG/HDL	≥4.06	0.85	85.0	71.1	68.8	86.4	2.95	0.21	56.1
		(0.832–0.868)	(82.3–87.5)	(68.2–73.9)	(66.6–71.0)	(84.2–88.3)	(2.66–3.27)	(0.18–0.25)	(52.1–59.9)
VAI	≥93.66	0.85	85.0	71.1	68.8	86.4	2.95	0.21	56.1
		(0.832–0.867)	(82.3–87.5)	(68.2–73.9)	(66.6–71.0)	(84.2–88.3)	(2.66–3.27)	(0.18–0.25)	(52.1–59.9)
SPISE	≤4.30	0.893	64.2	89.4	82.0	76.9	6.07	0.40	53.6
		(0.878–0.908)	(60.6–67.6)	(87.3–91.2)	(79.0–84.6)	(75.1–78.6)	(5.02–7.34)	(0.36–0.44)	(49.6–57.6)
BRI	≥4.41	0.841	84.2	69.4	67.3	85.4	2.76	0.23	53.6
		(0.822–0.859)	(81.4–86.8)	(66.4–72.2)	(65.1–69.5)	(83.2–87.4)	(2.50–3.04)	(0.19–0.27)	(49.6–57.4)
TyG	≥4.82	0.829	83.0	70.3	67.7	84.7	2.8	0.24	53.3
		(0.810–0.848)	(80.1–85.7)	(67.3–73.1)	(65.4–69.9)	(82.4–86.7)	(2.53–3.10)	(0.20–0.28)	(49.3–57.3)
ABSI	≥0.07	0.638	65.4	54.6	51.9	67.8	1.44	0.63	20.0
		(0.612–0.665)	(61.8–68.8)	(51.5–57.8)	(49.8–54.1)	(65.3–70.2)	(1.32–1.57)	(0.56–0.71)	(15.3–24.6)

IDF: International Diabetes Federation; AUC: area under the curve; Se: sensitivity; Sp: specificity; PPV: positive predictive value; NPV: negative predictive value; LRP: positive likelihood ratio; LRN: negative likelihood ratio; SPISE: single-point insulin sensitivity estimator; TG/HDL: triglyceride–HDL ratio; TyG: triglyceride–glucose index; LAP: lipid accumulation product; VAI: visceral adiposity index; METS-IR: metabolic score for insulin resistance; METS-VF: metabolic score for visceral fat; VAT: visceral adipose tissue; ABSI: body shape index; BRI: body rounding index.

**Table 9:** Accuracy measures of surrogate insulin resistance and obesity markers to identify metabolic syndrome in Mexican adult men aged 40–59 according to the IDF criteria. The sample consisted of 1034 subjects with *MetS* and 673 without *MetS*.

Index	Cut-Off Point	AUC (IC 95%)	Se (%)	Sp (%)	PPV (%)	NPV (%)	LRP	LRN	Youden Index
METS-IR	≥42.86	0.872	87.3	69.5	81.5	78.1	2.87	0.18	56.8
		(0.854–889)	(85.1–89.3)	(65.9–73.0)	(79.6–83.1)	(75.1–80.8)	(2.55–3.22)	(0.15–0.22)	(52.6–60.8)
LAP	≥59.07	0.867	86.7	69.6	81.4	77.3	2.86	0.19	56.3
		(0.849–885)	(84.5–88.7)	(66.0–73.1)	(79.6–83.1)	(74.4–80.1)	(2.55–3.22)	(0.16–0.22)	(52.2–60.1)
SPISE	≤4.44	0.863	65.4	86.4	88.1	61.9	4.84	0.40	51.8
		(0.845–0.881)	(62.4–68.3)	(83.6–88.9)	(85.9–90.0)	(59.8–64.0)	(3.97–5.88)	(0.37–0.44)	(47.8–55.5)
VAI	≥91.09	0.820	82.1	67.7	79.6	71.1	2.55	0.26	49.8
		(0.0.799–0.840)	(79.6–84.4)	(64.0–71.2)	(77.7–81.4)	(68.1–73.9)	(2.27–2.85)	(0.23–0.30)	(45.4–54.1)
VAT	≥1265.96	0.826	82.6	67.1	79.4	71.6	2.52	0.26	49.7
		(0.806–0.847)	(80.2–84.9)	(63.4–70.7)	(77.5–81.2)	(68.6–74.4)	(2.25–2.82)	(0.22–0.30)	(45.5–53.9)
TG/HDL	≥3.95	0.82	82.1	67.6	79.5	71.0	2.53	0.26	49.7
		(0.799–0.841)	(79.6–84.4)	(63.9–71.1)	(77.6–81.3)	(68.1–73.9)	(2.26–2.84)	(0.23–0.30)	(45.4–53.9)
METS-VF	≥7.14	0.826	82.5	67.1	79.4	71.5	2.52	0.26	49.6
		(0.806–0.847)	(80.1–84.8)	(63.4–70.7)	(77.5–81.2)	(68.5–74.3)	(2.25–2.81)	(0.22–0.30)	(45.3–53.8)
TyG	≥4.85	0.806	80.6	67.1	79.0	69.3	2.46	0.29	47.7
		(0.784–0.828)	(78.1–83.0)	(63.4–70.7)	(77.1–80.8)	(66.3–72.1)	(2.20–2.75)	(0.25–0.33)	(43.3–52.1)
BRI	≥4.98	0.792	79.3	64.9	77.6	67.1	2.26	0.32	44.2
		(0.769–0.815)	(76.7–81.7)	(61.1–68.5)	(75.7–79.4)	(64.1–69.9)	(2.03–2.52)	(0.28–0.36)	(39.7–48.8)
ABSI	≥0.08	0.596	61.0	50.8	65.5	45.9	1.24	0.77	11.8
		(0.569–0.624)	(57.9–64.0)	(46.9–54.6)	(63.5–67.6)	(43.2–48.5)	(1.13–1.36)	(0.69–0.85)	(7.0–16.6)

IDF: International Diabetes Federation; AUC: area under the curve; Se: sensitivity; Sp: specificity; PPV: positive predictive value; NPV: negative predictive value; LRP: positive likelihood ratio; LRN: negative likelihood ratio; SPISE: single-point insulin sensitivity estimator; TG/HDL: triglyceride–HDL ratio; TyG: triglyceride–glucose index; LAP: lipid accumulation product; VAI: visceral adiposity index; METS-IR: metabolic score for insulin resistance; METS-VF: metabolic score for visceral fat; VAT: visceral adipose tissue; ABSI: body shape index; BRI: body rounding index.

**Table 10:** Accuracy measures of surrogate insulin resistance and obesity markers to identify metabolic syndrome in Mexican adult men over 60 according to the IDF criteria. The sample consisted of 659 subjects with *MetS* and 446 without *MetS*.

Index	Cut-Off Point	AUC (95% CI)	Se (%)	Sp (%)	PPV (%)	NPV (%)	LRP	LRN	Youden Index
METS-IR	≥40.48	0.885	88.6	71.5	82.1	80.9	3.11	0.16	60.1
		(0.864–0.905)	(85.9–90.9)	(67.0–75.6)	(79.8–84.2)	(77.3–84.1)	(2.68–3.61)	(0.13–0.20)	(55.2–64.9)
LAP	≥45.81	0.891	89.2	70.4	81.6	81.5	3.01	0.15	59.6
		(0.872–0.910)	(86.6–91.4)	(65.9–74.6)	(79.3–83.7)	(77.8–84.7)	(2.61–3.49)	(0.12–0.19)	(54.8–64.4)
METS-VF	≥7.37	0.833	83.4	69.2	80.0	73.9	2.72	0.24	52.6
		(0.808–0.859)	(80.4–86.2)	(64.7–73.5)	(77.6–82.2)	(70.2–77.2)	(2.35–3.14)	(0.20–0.29)	(47.5–57.9)
VAT	≥1603.28	0.833	83.4	69.2	80.0	73.9	2.72	0.24	52.6
		(0.808–0.859)	(80.4–86.2)	(64.7–73.5)	(77.6–82.2)	(70.2–77.2)	(2.35–3.14)	(0.20–0.29)	(47.5–57.9)
VAI	≥80.01	0.825	82.7	69.5	80.0	73.1	2.71	0.25	52.2
		(0.799–0.850)	(79.5–85.5)	(65.0–73.7)	(77.6–82.2)	(69.4–76.4)	(2.35–3.13)	(0.21–0.30)	(46.9–57.3)
TG/HDL	≥3.46	0.825	82.7	69.2	79.9	73.0	2.69	0.25	51.9
		(0.799–0.850)	(79.5–85.5)	(64.7–73.5)	(77.5–82.1)	(69.4–76.4)	(2.33–3.11)	(0.21–0.30)	(46.8–57.1)
TyG	≥4.75	0.824	82.5	68.8	79.6	72.7	2.65	0.25	51.3
		(0.799–0.849)	(79.4–85.3)	(64.3–73.1)	(77.2–81.8)	(69.0–76.1)	(2.30–3.05)	(0.21–0.30)	(46.2–56.6)
SPISE	≤ 4.88	0.875	63.1	87.6	88.3	61.6	5.12	0.42	50.7
		(0.854–0.896)	(59.3–66.8)	(84.2–90.5)	(85.4–90.7)	(59.1–64.1)	(3.97–6.60)	(0.38–0.47)	(45.9–55.5)
BRI	≥5.13	0.806	80.7	67.9	78.8	70.4	2.52	0.28	48.6
		(0.779–0.834)	(77.5–83.6)	(63.3–72.2)	(76.3–81.0)	(66.8–73.8)	(2.19–2.90)	(0.24–0.34)	(43.3–53.7)
ABSI	≥0.08	0.597	61.1	53.8	66.1	48.3	1.32	0.72	14.9
		(0.563–0.632)	(57.3–64.8)	(49.0–58.5)	(63.5–68.7)	(45.1–51.6)	(1.18–1.49)	(0.63–0.82)	(9.1–20.7)

IDF: International Diabetes Federation; AUC: area under the curve; Se: sensitivity; Sp: specificity; PPV: positive predictive value; NPV: negative predictive value; LRP: positive likelihood ratio; LRN: negative likelihood ratio; SPISE: single-point insulin sensitivity estimator; TG/HDL: triglyceride–HDL ratio; TyG: triglyceride–glucose index; LAP: lipid accumulation product; VAI: visceral adiposity index; METS-IR: metabolic score for insulin resistance; METS-VF: metabolic score for visceral fat; VAT: visceral adipose tissue; ABSI: body shape index; BRI: body rounding index.

**Table 11:** Accuracy measures of surrogate insulin resistance and obesity markers to identify metabolic syndrome in Mexican adult women aged 20–39 according to the IDF criteria. The sample consisted of 1017 subjects with *MetS* and 1267 without *MetS*.

Index	Cut-Off Point	AUC (95% CI)	Se (%)	Sp (%)	PPV (%)	NPV (%)	LRP	LRN	Youden Index
TyG	≥4.67	0.898	89.8	73.3	73.0	90.0	3.37	0.14	63.1
		(0.885–0.911)	(87.8–91.6)	(70.8–75.7)	(71.1–74.8)	(88.2–91.5)	(3.07–3.70)	(0.11–0.17)	(60.1–66.1)
LAP	≥46.16	0.903	90.3	71.1	71.5	90.2	3.14	0.14	61.4
		(0.891–0.915)	(88.3–92.1)	(68.6–73.6)	(69.7–73.3)	(88.3–91.7)	(2.87–3.43)	(0.11–0.16)	(58.2–64.4)
VAI	≥91.42	0.904	90.4	70.0	70.8	90.1	3.02	0.14	60.4
		(0.892–0.917)	(88.4–92.2)	(67.4–72.6)	(69.0–72.5)	(88.3–91.7)	(2.77–3.30)	(0.11–0.17)	(57.3–63.4)
TG/HDL	≥2.90	0.905	90.5	69.8	70.6	90.2	3.00	0.14	60.3
		(0.892–0.917)	(88.6–92.2)	(67.2–72.3)	(68.8–72.4)	(88.3–91.8)	(2.76–3.27)	(0.11–0.16)	(57.0–63.3)
SPISE	≤4.56	0.848	66.3	84.9	77.9	75.8	4.40	0.40	51.2
		(0.832–0.863)	(63.3–69.2)	(82.8–86.8)	(75.4–80.2)	(74.2–77.4)	(3.83–5.05)	(0.36–0.43)	(47.8–54.7)
METS-IR	≥42.18	0.835	83.5	66.8	66.9	83.5	2.52	0.25	50.3
		(0.819–0.851)	(81.1–85.8)	(64.1–69.4)	(65.0–68.7)	(81.4–85.4)	(2.32–2.74)	(0.21–0.28)	(46.8–53.7)
VAT	≥1301.41	0.786	78.6	62.6	62.8	78.5	2.11	0.34	41.2
		(0.767–0.804)	(76.0–81.1)	(59.9–65.3)	(61.0–64.6)	(76.3–80.5)	(1.95–2.28)	(0.30–0.39)	(37.4–44.8)
METS-VF	≥7.17	0.786	78.6	62.6	62.8	78.5	2.11	0.34	41.2
		(0.767–0.804)	(76.0–81.1)	(59.9–65.3)	(61.0–64.6)	(76.3–80.5)	(1.95–2.28)	(0.30–0.39)	(37.4–44.8)
BRI	≥4.99	0.751	75.2	61.2	60.9	75.4	1.94	0.4	36.4
		(0.731–0.770)	(72.4–77.8)	(58.5–63.9)	(59.0–62.7)	(73.2–77.5)	(1.80–2.10)	(0.36–0.45)	(32.6–40.1)
ABSI	≥0.07	0.569	58.0	52.4	49.5	60.9	1.22	0.8	10.4
		(0.546–0.593)	(54.9–61.0)	(49.6–55.2)	(47.5–51.4)	(58.7–63.0)	(1.13–1.32)	(0.73–0.87)	(6.4–14.4)

IDF: International Diabetes Federation; AUC: area under the curve; Se: sensitivity; Sp: specificity, PPV: positive predictive value; NPV: negative predictive value; LRP: positive likelihood ratio; LRN: negative likelihood ratio; SPISE: single-point insulin sensitivity estimator; TG/HDL: triglyceride–HDL ratio; TyG: triglyceride–glucose index; LAP: lipid accumulation product; VAI: visceral adiposity index; METS-IR: metabolic score for insulin resistance; METS-VF: metabolic score for visceral fat; VAT: visceral adipose tissue; ABSI: body shape index; BRI: body rounding index.

**Table 12:** Accuracy measures of surrogate insulin resistance and obesity markers to identify metabolic syndrome in Mexican adult women aged 40–59 according to the IDF criteria. The sample consisted of 1659 subjects with *MetS* and 729 without *MetS*.

Index	Cut-Off Point	AUC (95% CI)	Se (%)	Sp (%)	PPV (%)	NPV (%)	LRP	LRN	Youden Index
TyG	≥4.73	0.893	89.3	74.7	88.9	75.5	3.54	0.14	64.0
		(0.878–0.907)	(87.8–90.8)	(71.4–77.8)	(87.6–90.1)	(72.8–78.1)	(3.12–4.02)	(0.12–0.16)	(60.5–67.4)
LAP	≥55.94	0.875	87.5	70.1	86.9	71.2	2.93	0.18	57.6
		(0.860–0.891)	(85.9–89.1)	(66.6–73.4)	(85.6–88.1)	(68.4–73.9)	(2.62–3.28)	(0.15–0.20)	(53.9–61.2)
VAI	≥87.28	0.892	89.2	68.0	86.4	73.5	2.79	0.16	57.2
		(0.879–0.906)	(87.6–90.7)	(64.5–71.4)	(85.1–87.6)	(70.6–76.3)	(2.51–3.11)	(0.14–0.18)	(53.5–60.9)
TG/HDL	≥2.76	0.893	89.3	67.7	86.3	73.7	2.77	0.16	57.0
		(0.879–0.906)	(87.8–90.8)	(64.2–71.1)	(85.0–87.5)	(70.7–76.5)	(2.49–3.08)	(0.13–0.18)	(53.2–60.7)
METS-IR	≥43.69	0.816	81.6	65.9	84.5	61.2	2.4	0.28	47.5
		(0.797–0.835)	(79.7–83.5)	(62.4–69.4)	(83.1–85.8)	(58.5–63.9)	(2.16–2.66)	(0.25–0.31)	(43.5–51.3)
SPISE	≤ 4.44	0.812	62.9	81.3	88.5	49.0	3.37	0.46	44.2
		(0.793–0.831)	(60.5–65.3)	(78.3–84.1)	(86.8–90.0)	(47.2–50.8)	(2.89–3.94)	(0.42–0.49)	(40.5–47.9)
VAT	≥2007.23	0.746	74.6	60.9	81.3	51.3	1.91	0.42	35.5
		(0.723–0.768)	(72.5–76.7)	(57.2–64.4)	(79.8–82.7)	(48.8–53.9)	(1.74–2.10)	(0.38–0.46)	(31.3–39.5)
METS-VF	≥7.60	0.746	74.6	60.9	81.3	51.3	1.91	0.42	35.5
		(0.723–0.768)	(72.5–76.7)	(57.2–64.4)	(79.8–82.7)	(48.8–53.9)	(1.74–2.10)	(0.38–0.46)	(31.3–39.5)
BRI	≥5.70	0.706	70.7	59.6	79.9	47.2	1.75	0.49	30.3
		(0.683–0.730)	(68.4–72.8)	(56.0–63.2)	(78.4–81.4)	(44.8–49.6)	(1.60–1.93)	(0.45–0.54)	(26.1–34.3)
ABSI	≥0.08	0.565	57.5	51.8	73.1	34.9	1.19	0.82	9.3
		(0.540–0.590)	(55.0–59.9)	(48.1–55.5)	(71.3–74.7)	(32.9–36.9)	(1.10–1.30)	(0.75–0.90)	(4.8–13.0)

IDF: International Diabetes Federation; AUC: area under the curve; Se: sensitivity; Sp: specificity; PPV: positive predictive value; NPV: negative predictive value; LRP: positive likelihood ratio; LRN: negative likelihood ratio; SPISE: single-point insulin sensitivity estimator; TG/HDL: triglyceride–HDL ratio; TyG: triglyceride–glucose index; LAP: lipid accumulation product; VAI: visceral adiposity index; METS-IR: metabolic score for insulin resistance; METS-VF: metabolic score for visceral fat; VAT: visceral adipose tissue; ABSI: body shape index; BRI: body rounding index.

**Table 13:** Accuracy measures of surrogate insulin resistance and obesity markers to identify metabolic syndrome in Mexican adult women over 60 according to the IDF criteria. The sample consisted of 948 subjects with *MetS* and 421 without *MetS*.

Index	Cut-Off Point	AUC (95% CI)	Se (%)	Sp (%)	PPV (%)	NPV (%)	LRP	LRN	Youden Index
TyG	≥4.74	0.848	84.9	73.6	87.8	68.4	3.22	0.2	58.5
		(0.826–0.871)	(82.4–87.1)	(69.1–77.7)	(86.0–89.5)	(64.8–71.8)	(2.74–3.79)	(0.17–0.24)	(53.8–63.2)
LAP	≥48.70	0.881	88.1	66.0	85.3	71.2	2.6	0.18	54.1
		(0.863–0.899)	(85.9–90.1)	(61.2–70.5)	(83.6–87.0)	(67.3–74.9)	(2.27–2.97)	(0.15–0.22)	(49.1–59.0)
TG/HDL	≥2.78	0.837	83.7	66.9	85.1	64.6	2.54	0.24	50.6
		(0.815–0.860)	(81.2–86.0)	(62.2–71.4)	(83.2–86.7)	(60.9–68.2)	(2.21–2.91)	(0.21–0.28)	(45.5–55.0)
VAI	≥87.30	0.837	83.7	66.9	85.1	64.6	2.54	0.24	50.6
		(0.815–0.859)	(81.2–86.0)	(62.2–71.4)	(83.2–86.7)	(60.9–68.2)	(2.21–2.91)	(0.21–0.28)	(45.5–55.0)
METS-IR	≥40.80	0.81	81.1	66.2	84.4	60.9	2.4	0.28	47.3
		(0.785–0.836)	(78.4–83.5)	(61.5–70.7)	(82.5–86.1)	(57.3–64.3)	(2.10–2.76)	(0.25–0.33)	(42.0–52.5)
SPISE	≤ 4.81	0.803	61.2	80.5	87.6	48.0	3.14	0.48	41.7
		(0.777–0.830)	(58.1–64.4)	(76.4–84.2)	(85.2–89.6)	(45.7–50.3)	(2.57–3.84)	(0.44–053)	(36.7–46.5)
VAT	≥2539.43	0.756	75.7	64.1	82.6	54.0	2.11	0.38	39.8
		(0.727–0.786)	(72.8–78.4)	(59.3–68.7)	(80.6–84.4)	(50.6–57.2)	(1.85–2.41)	(0.33–0.43)	(34.4–45.1)
METS-VF	≥7.83	0.756	75.7	64.1	82.6	54.0	2.11	0.38	39.8
		(0.727–0.786)	(72.8–78.4)	(59.3–68.7)	(80.6–84.4)	(50.6–57.2)	(1.85–2.41)	(0.33–0.43)	(34.4–45.1)
BRI	≥5.94	0.724	72.4	60.5	80.5	49.4	1.84	0.45	32.9
		(0.693–0.756)	(69.5–75.2)	(55.7–65.2)	(78.5–82.4)	(46.2–52.6)	(1.62–2.08)	(0.40–0.52)	(27.4–38.3)
ABSI	≥0.08	0.602	61.1	55.8	75.7	38.9	1.38	0.70	16.9
		(0.569–0.635)	(58.0–64.3)	(50.9–60.6)	(73.4–77.8)	(36.2–41.7)	(1.23–1.56)	(0.62–0.78)	(11.1–22.5)

IDF: International Diabetes Federation; AUC: area under the curve; Se: sensitivity; Sp: specificity; PPV: positive predictive value; NPV: negative predictive value; LRP: positive likelihood ratio; LRN: negative likelihood ratio; SPISE: single-point insulin sensitivity estimator; TG/HDL: triglyceride–HDL ratio; TyG: triglyceride–glucose index; LAP: lipid accumulation product; VAI: visceral adiposity index; METS-IR: metabolic score for insulin resistance; METS-VF: metabolic score for visceral fat; VAT: visceral adipose tissue; ABSI: body shape index; BRI: body rounding index.

## Data Availability

The data presented in this study are openly available at https://ensanut.insp.mx/ (accessed on 19 November 2021).

## Appendix A

The diagnostic criteria of the National Cholesterol Education Program Adult Treatment Panel III (NCEP ATPIII) and the IDF are shown in Table A1.

**Table A1:** Definitions of *MetS* according to the NCEP ATP III and the IDF.

Definition	ATP III	IDF
Criteria	Central obesity and any 2 out of these 4 other factors	≥3 out of these parameters
Waist Circumference	≥102 cm (men), ≥88 cm (women)	≥90 cm (men), ≥80 cm (women)
Triglycerides	≥150 mg/dL or drug treatment for elevated levels	≥150 mg/dL or drug treatment for elevated levels
HDL Cholesterol	<40 mg/dL (men), <50 mg/dL (women) or drug treatment for elevated levels	<40 mg/dL (men), <50 mg/dL (women) or drug treatment for elevated levels
Blood Pressure	≥130/85 mmHg or drug treatment for high blood pressure	≥130/85 mmHg dL or drug treatment for high blood pressure
Fasting Glucose	≥110 mg/dL or drug treatment for diabetes mellitus	≥100 mg/dL or drug treatment for diabetes mellitus
